# Self‐Care Interventions for Preventing Cardiovascular Diseases After Hypertensive Pregnancy Disorders: A Systematic Review and Meta‐Analysis

**DOI:** 10.1111/1471-0528.18152

**Published:** 2025-03-28

**Authors:** Thuy D. T. Mai, Sho Katsuragawa, Annie McDougall, Phi‐Yen Nguyen, Lorena Romero, Joshua Vogel, Maureen Makama

**Affiliations:** ^1^ Women's, Children's and Adolescents' Health Program Burnet Institute Melbourne Victoria Australia; ^2^ The Ian Potter Library The Alfred Melbourne Victoria Australia

**Keywords:** cardiovascular diseases, hypertensive disorders of pregnancy, self‐care interventions

## Abstract

**Background:**

Women with previous hypertensive disorders of pregnancy (HDP) have an increased risk of developing cardiovascular diseases (CVDs) later in life. Self‐care interventions are known to promote health and well‐being, such as self‐measured blood pressure or mindfulness interventions.

**Objectives:**

We evaluated whether self‐care interventions reduced the incidence of cardiovascular events, chronic hypertension, and the risk of CVDs in women with previous HDP.

**Search Strategy:**

MEDLINE, Embase, CINAHL, PsycINFO, and the Cochrane Library were systematically searched on 08 January 2025 without date or language restrictions.

**Selection Criteria:**

Randomised and non‐randomised controlled trials.

**Data Collection and Analysis:**

Meta‐analysis was performed using random‐effects models.

**Main Results:**

We included 16 studies involving 2200 women. Fourteen studies from twelve randomised trials, one was a non‐randomised trial, and one was a quasi‐experimental study. Data from nine trials involving 952 women with low‐certainty evidence showed that self‐care interventions may be associated with lower systolic (mean difference from baseline (MD) −3.24; 95% CI −5.42, −1.06 mmHg) and diastolic (MD −3.07; 95% CI −4.88, −1.25 mmHg) blood pressure. Self‐care interventions likely decrease the risk of postpartum hypertension readmission (RR 0.35; 95% CI 0.14, 0.89; 3 trials; 605 women; moderate‐certainty evidence). There were insufficient studies to pool results for cardiovascular events and chronic hypertension.

**Conclusions:**

There was limited evidence to support a recommendation for using self‐care interventions to prevent CVDs in women with previous HDP, although some self‐care interventions may reduce blood pressure and the risk of postpartum hypertension readmission. Larger trials with multiple and longer follow‐ups utilising the core outcome set of CVDs are needed.

## Abstract 2

**Linked article:** This article is commented on by Merriel pp. 1362–1363 in this issue. To view this article visit https://doi.org/10.1111/1471‐0528.18232.

## 1 Introduction

Hypertensive disorders of pregnancy (HDP) refer to a group of conditions, including chronic (preexisting) hypertension, preeclampsia/eclampsia, and gestational hypertension [1]. Globally HDP affects 18.1 million women yearly [2], and its burden is greatest in low‐ and middle‐income countries (LMICs), accounting for 95% of HDP maternal deaths worldwide [3]. Women who experienced HDP are at a two‐to four‐fold higher risk of developing cardiovascular diseases (CVDs) in later life, including hypertension (relative risk (RR) 3.46; 95% confidence interval (CI) 2.67, 4.49) or heart failure (RR 2.53; 95% CI 1.28, 5.00) [4]. These elevated risks may persist for at least a decade [5, 6, 7], and even after they have developed, CVDs can be a silent or asymptomatic condition for many years [8, 9, 10]. CVDs encompass heart and blood vessel disorders, such as coronary heart disease, rheumatic heart disease, and deep vein thrombosis [11]. CVDs are among the leading causes of death globally, at least 75% of which occur in LMICs, accounting for 9.2 million female deaths in 2019 [12, 13, 14].

Women with a history of HDP may benefit from interventions aimed at reducing CVD risk factors. In recent years, the World Health Organisation (WHO) has promoted self‐care interventions as a promising approach to enhance people's health and well‐being [15]. Self‐care interventions can include tools to support and actions to improve individuals' ability to care for themselves [16]. Self‐care interventions encompass various approaches, including health literacy, regular physical activity, understanding and practices to mitigate risks, awareness and taking action against dangers [17].

For chronic disease prevention and management, self‐care interventions usually relate to behavioural change, equipping people with the knowledge and skills needed to be responsible and actively self‐maintain, self‐monitor, and self‐manage their illness [18]. One commonly proposed mechanism suggests that self‐care actions have a cardioprotective effect, such as minimising inflammation or avoiding risky pharmacological treatment [19], serving as complementary interventions alongside primary treatments for underlying conditions. For example, maintaining a high‐fibre diet helps improve cholesterol levels and endothelial function to decrease inflammation, a risk factor for CVDs [20, 21].

Early prevention strategies with self‐care actions, including practices, lifestyle choices, and habits, might be effective in mitigating the risk of developing CVDs in women with previous HDP. Although there are existing reviews on different interventions to reduce CVD risks in this population, such as lifestyle interventions or home blood pressure (BP) monitoring [22, 23, 24, 25, 26], no reviews have specifically evaluated all components of self‐care interventions together. Furthermore, evidence is absent on the effectiveness of recommended self‐care interventions for women with a history of HDP [27]; it is crucial to identify whether self‐care interventions are effective in preventing CVD risks and outcomes for this high‐risk population. This study aimed to systematically evaluate the evidence on the effects of self‐care interventions on the incidence of cardiovascular events chronic hypertension and risk factors of CVDs in women with a history of HDP.

## 2 Methods

This systematic review was conducted following the Preferred Reporting Items for Systematic Reviews and Meta‐Analyses (PRISMA) guidelines (Appendix S1) [28]. The protocol was prospectively registered with PROSPERO (CRD42023473686) (Appendix S9).

### 2.1 Eligibility Criteria

Primary randomised and non‐randomised trials (including cross‐over trials, cluster randomised and quasi‐experimental trials) administering self‐care interventions to women with a history of HDP were eligible. There were no limits based on duration since the last pregnancy. To ensure a homogeneous study population and better isolate the impact of self‐care interventions, studies including women with preexisting CVDs (such as stroke, renal failure, or cardiac disease) before the index pregnancy were excluded. Caregivers (family members or partners) carrying out self‐care for women with HDP were excluded. We defined a self‐care intervention as any non‐pharmacological strategy, tool, or resource designed to encourage or support self‐care. The intervention could be single or combined with other self‐care interventions to increase the coverage and quality of healthcare services and/or enhance well‐being, health, and care experiences. Eligible comparisons were no intervention, usual or standard care. Trials were not eligible if their primary interest was in another population, such as gestational diabetes or high BMI, even though they reported on women with previous HDP. There were no restrictions on the language or date of publication.

### 2.2 Information Sources and Search Strategy

Studies were retrieved from MEDLINE, Embase, CINAHL, PsycINFO, and the Cochrane Library on 09 November 2023, and updated on 08 January 2025 using a pre‐defined search strategy developed in consultation with an information specialist (LR) (Appendix S2). Each electronic database was searched using adapted free text and index terms for the two main concepts: (a) hypertensive disorders of pregnancy and (b) self‐care interventions. Additionally, we manually reviewed the reference lists of included studies to identify any other relevant studies.

### 2.3 Study Selection and Research Integrity Assessment

Citations were collated using EndNote X9 [29], and screening was conducted using Covidence [30]. After removing duplicates, two reviewers (TM and SK) independently assessed the titles and abstracts, followed by the full text of the remaining citations to determine eligibility. Any disagreements were resolved through discussion and consultation with a third reviewer (MM or AM). Google Translate was used for articles published in languages other than English.

We contacted authors as required to obtain further information for (a) research integrity assessment (RIA), (b) data extraction and analysis, such as missing values needed for meta‐analysis, and (c) clarification of data. Studies for which authors did not respond after two attempts to contact them via email to obtain sufficient data for analysis were recorded as no response. All eligible studies underwent RIA using a published RIA tool [31] by two independent reviewers (TM and SK).

### 2.4 Data Extraction and Quality Assessment

Two reviewers (TM and SK) independently extracted data from eligible studies using Covidence [30]. Details extracted included study characteristics, design, sample size, duration of study enrolment, participant characteristics, duration and type of self‐care intervention, and the primary and secondary outcomes reported. Review outcomes were selected based on the recommended core outcome set of CVD [32, 33, 34] and commonly used outcomes from reviews on interventions for people with HDP [22, 25, 26]. The primary outcomes were (1) the incidence of any cardiovascular, event (such as stroke, myocardial infarction, or heart failure) and (2) chronic hypertension. Risk factors and additional secondary outcomes are BMI, diabetes, BP, lipid levels, physical activity, dietary intake, breastfeeding, psychological issues, smoking, caffeine use, alcohol use, illicit drug use status, social harms autonomy, and antihypertensive medication requirement (Appendix S3). Studies were not excluded based on review outcomes. We extracted all studies' characteristics, including those that did not report review outcomes. Any discrepancies were resolved through discussion.

The quality of identified randomised controlled trials (RCTs) was assessed using the revised Cochrane risk‐of‐bias tool for randomised trials (RoB 2) [35], and for non‐randomised trials (non‐RCTs), the risk‐of‐bias in non‐randomised studies of interventions (ROBINS‐I) tool [36]. Two reviewers independently assessed each study (TM and SK), and any disagreements were resolved by a third reviewer (MM or AM). Risk‐of‐bias plots were presented using Robvis [37]. Two reviewers (TM and SK) independently assessed the certainty of evidence using Grading of Recommendations, Assessment, Development, and Evaluations (GRADE) [38] in GRADEpro [39]. Disagreements were resolved through discussion or consultation with a third reviewer (MM or AM).

### 2.5 Data Synthesis

Studies' characteristics were reported descriptively. Data analysis was carried out using STATA SE 18 [40]. For outcomes reported by at least 2 studies, a meta‐analysis was conducted [41]. Results from non‐RCTs were not included in a meta‐analysis. Findings from non‐RCTs and outcomes reported by < 2 trials are presented narratively. Statistical heterogeneity was assessed with a random‐effects model due to the potential sources of variations in intervention effects and sampling error across trials [41]. Heterogeneity was considered substantial if I^2^ > 50% [41]. Meta‐analyses were presented as RR (for binomial variables) or mean difference (MD) from baseline (for continuous variables), their 95% CI, and I^2^ values. Publication bias assessment via funnel plots or statistical tests was not completed as no outcomes were reported by 10 or more studies [38]. Descriptions of dealing with missing data, the unit of analysis issues, sensitivity analyses, sub‐group analyses, investigation of heterogeneity, and post hoc analyses are detailed in Appendix S4.

## 3 Results

### 3.1 Study Selection

**FIGURE 1:**
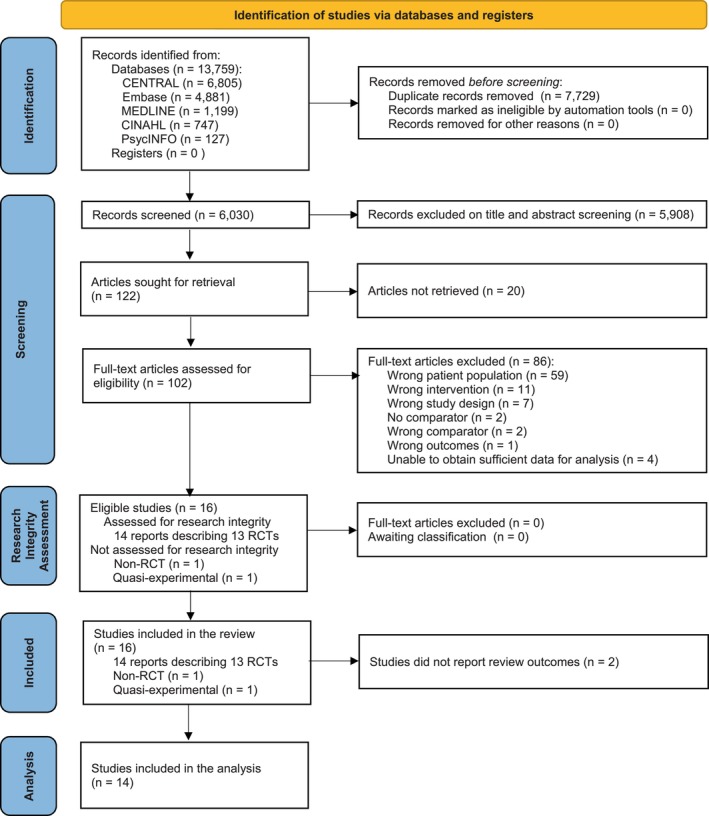
PRISMA flow chart of included studies. The research integrity tool was designed to assess RCTs only; therefore, non‐RCTs did not undergo research integrity assessment.

The search identified 13 759 records. After removing duplicates, 6030 unique records were eligible for title and abstract screening. Of the 122 articles sought for full‐text retrieval, 102 studies were available in full text, with 20 unable to be sourced. There were 16 studies included in this review (14 studies describing 13 RCTs [42, 43, 44, 45, 46, 47, 48, 49, 50, 51, 52, 53, 54, 55], one non‐RCT [56], and one quasi‐experimental study [57]). Two studies did not report review outcomes, leaving 14 included in the analysis (Figure 1 and Appendix S5). No studies were excluded from the analysis based on RIA (Appendix S6).

### 3.2 Study Characteristics

The review included 16 studies involving 2200 participants and reporting self‐care interventions for women with previous HDP [42, 43, 44, 45, 46, 47, 48, 49, 50, 51, 52, 53, 56, 57]. One study [53] was a secondary analysis of another trial [50]. All studies were published between 2018 and 2024 and were conducted in eight countries – the United States (*n* = 8) [44, 45, 48, 49, 51, 52, 55, 56], England (*n* = 3) [50, 53, 54], Australia (*n* = 1) [43], Canada (*n* = 1) [47], and Germany (*n* = 1) [46], the Netherlands and Germany (*n* = 1) [42], and a lower‐middle‐income country Indonesia (*n* = 1) (Table 1) [57].

**TABLE 1:** Characteristics of the included studies.

Author Year	Study design	Country	Intervention vs. Control^a^	Postpartum period at enrolment	Intervention duration	Sample size^b^	Review outcomes^c^
Arkerson 2023	RCT	USA	HBPM vs. IBPM	Soon after discharge	16 days	197	Additional visits, medication
Cairns 2018	RCT	England	HBPM vs. Usual care	Soon after discharge	6 months	82	BP, medication, Psychological status
Ekawati 2019	Non‐RCT^d^	Indonesia	Lifestyle change vs. Not specified	On the 2nd day	7 days	60	No outcomes of interest
Hauspurg 2023	RCT	USA	HBPM vs. HBPM+ Lifestyle change vs. Usual care	6 weeks to 6 months	12 months	129	BP, BMI, medication, Hypertension, PA, Retention, Self‐efficacy
Hirshberg 2018	RCT	USA	HBPM vs. IBPM	4 to 6 days	2 weeks	206	Additional visits, medication
Hoppe 2020	Non‐RCT	USA	HBPM vs. Standard care	Soon after discharge	6 weeks	428	Additional visits, medication
Hutchesson 2020	RCT	Australia	Lifestyle change vs. Usual care	Within 4 years	3 months	31	BP, BMI, Dietary intake, Lipid levels, PA, Psychological status
Kitt 2021	RCT	England	HBPM vs. Usual care	Soon after discharge	6 months	61	BP
Kitt 2023	RCT	England	HBPM vs. Standard care	1 to 6 days	9 months	200	BP, BMI, medication, Readmission, PA, Smoking, Breastfeeding
Lewey 2022	RCT	USA	PA vs. No intervention	1 to 4 months	3 months	127	BP, PA
Muijsers 2022	RCT	Netherlands, Germany	HBPM vs. Usual care	At least 1 year	12 months	191	BP, BMI, medication, Chest pain, Diabetes, Hypertension, Smoking
Nicklas 2024	RCT	USA	Lifestyle change vs. Usual care	1 to 3 months	6 weeks	58^e^	BP, BMI, Dietary intake, Lipid levels, PA, Psychological status
Parfenova 2021	RCT	Canada	Lifestyle change vs. Usual care	1 to 18 months	1 month	98	No outcomes of interest
Rich‐Edwards 2019	RCT	USA	Lifestyle change vs. Usual care	Within 5 years	9 months	139^d^	PA, Self‐efficacy
Riemer 2021	RCT	Germany	Lifestyle change vs. Usual care	6 weeks	6 months	29	BP
Wang 2024	RCT	USA	Neonatal sleep vs. Usual care	6 weeks	4 months	104^e^	BP, medication, Hypertension

Abbreviations: Additional visits: Hypertension readmission and Additional emergency department or office visits for hypertension which did not result in readmission; BMI: body mass index; BP: blood pressure; HBPM: home blood pressure monitoring; Hypertension: Stage 1 or greater HTN or on hypertensives; IBPM: In‐office blood pressure monitoring; medication: use of antihypertensives; PA: Physical activity; RCT: randomised controlled trial.

^a^ Descriptions of self‐care interventions are detailed in Appendix S6.

^b^ Sample size included in the analysis.

^c^ No studies reported on review outcomes of interest: caffeine, illicit drug use status, and social harms.

^d^ Quasi‐experimental study.

^e^ Sample size at the last follow‐up.

The components of self‐care interventions included home‐based BP monitoring (*n* = 8) [42, 48, 49, 50, 51, 53, 54, 56]; lifestyle change (*n* = 6) [43, 46, 47, 52, 55, 57]; physical exercise (*n* = 1) [45]; neonatal sleep (*n* = 1) [44]. Control groups received usual care (*n* = 10) [42, 43, 44, 46, 47, 49, 50, 52, 53, 55]; standard care (*n* = 2), in‐office BP (*n* = 2) [48, 51], no intervention (*n* = 1) [45], and not specified (*n* = 1) [57]. The time of enrolment into the study ranged from post‐delivery to 5 years postpartum. Most studies implemented self‐care interventions during the first 6a months postpartum (*n* = 12) [44, 45, 46, 47, 48, 49, 50, 51, 53, 55, 56, 57]. The most commonly reported outcomes were systolic and diastolic BP (*n* = 10) [42, 44, 45, 46, 49, 50, 53, 54, 55, 58] and the use of antihypertensives (*n* = 7) (Table 1) [42, 44, 48, 49, 50, 51, 54]. Descriptions of self‐care intervention in each study arm, the number of participants at baseline, and attrition are detailed in Appendix S6.

### 3.3 Risk of Bias of Included Studies

Twelve studies from eleven RCTs had a high risk of bias primarily due to missing outcome data [43, 44, 46, 47, 49, 50, 51, 52, 53, 55], and bias in the measurement of the outcome [47, 48, 51, 52]. One RCT had some concerns [45], and one RCT had a low risk of bias (Appendix S7, Figure S1) [54]. One non‐RCT had a critical risk of bias primarily due to the risk of confounding [57], and the other had a serious risk of bias due to confounding and outcome measurements (Appendix S7, Figure S2) [56].

### 3.4 Synthesis of Results

Results from twelve studies reported eleven RCTs [42, 43, 44, 45, 46, 47, 48, 49, 50, 51, 52, 53, 54, 55] were included in the meta‐analyses. One study had two intervention arms; hence, the data were combined and considered as a single intervention arm [49]. Seven studies did not report the SDs for change in outcome measures [42, 44, 45, 46, 50, 52, 54], and were calculated from the reported baseline and final SD values.

### 3.5 Primary Outcomes – Cardiovascular Events and Chronic Hypertension

#### 3.5.1 Chest Pain

One trial involving 198 women found no association between self‐care interventions and the risk of chest pain (RR 0.67; 95% CI 0.30, 1.48; *p* = 0.31) [42].

#### 3.5.2 Stage 1 or Greater Hypertension or on Antihypertensive Medications (130/80 mmHg or Higher)

One trial involving 76 women reported no association between self‐care interventions and the incidence of the composite outcome stage 1 or greater hypertension or antihypertensive medications (RR 0.76, 95% CI 0.50, 1.16) [44]. Similarly, another trial involving 129 women reported no association (RR 1.26, 95% CI 0.91, 1.75) [49].

#### 3.5.3 Stage 2 Hypertension and/or on Antihypertensive Medications (140/90 mmHg or Higher)

One trial involving 129 women reported no association between self‐care interventions and the incidence of the composite outcome stage 2 hypertension and/or on antihypertensive medication (RR 1.20; 95% CI 0.58, 2.48; *p* = 0.61) [49].

### 3.6 Secondary Outcomes

**FIGURE 2:**
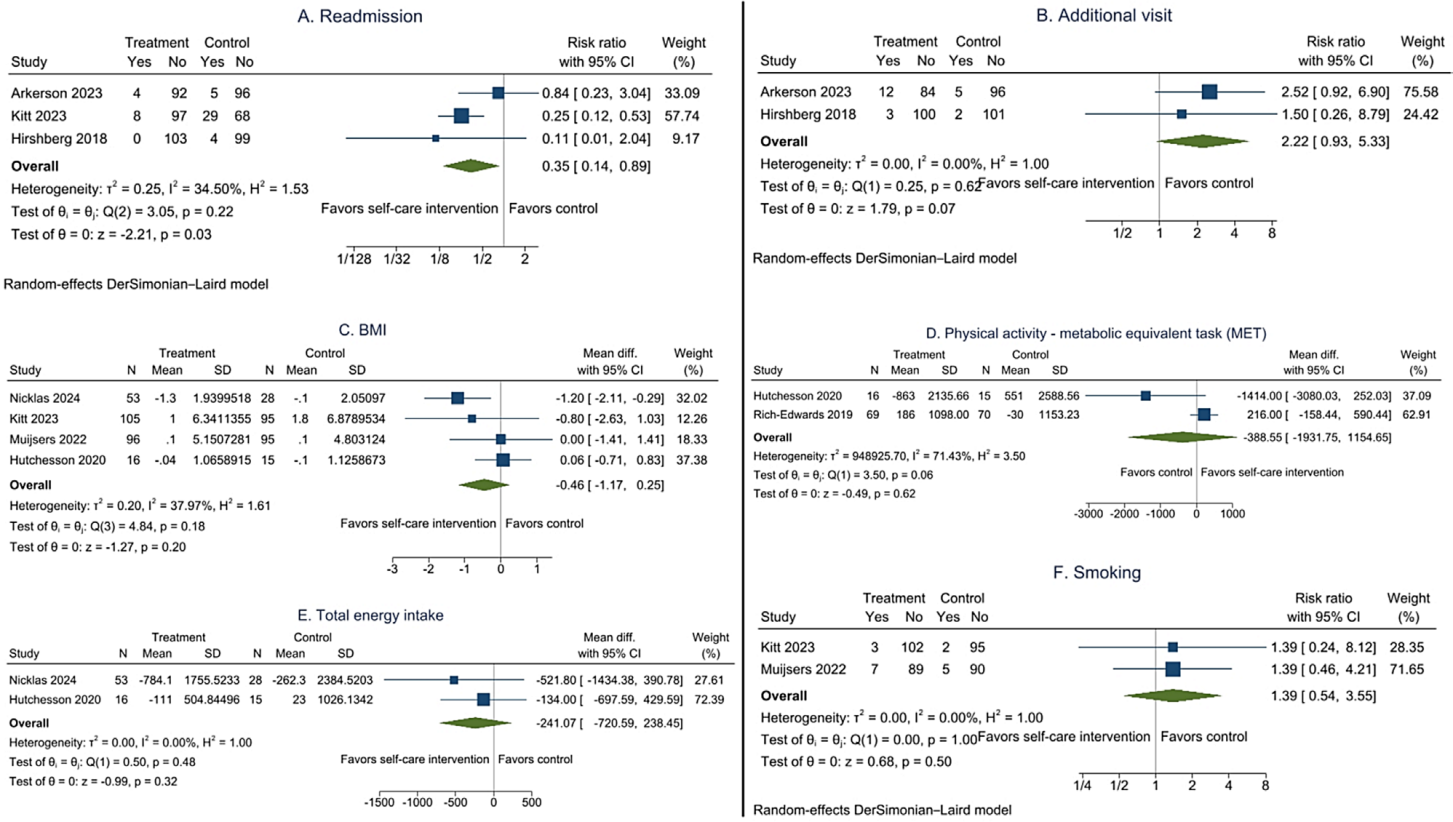
The effects of self‐care interventions on the mean difference from baseline of systolic blood pressure (A) and diastolic blood pressure (B), and the subgroup analysis by duration for systolic blood pressure (C) and diastolic blood pressure (D).

Data from 9 trials involving 952 women showed that compared to women in the control group, women receiving self‐care interventions may have lower systolic (MD from baseline −3.24; 95% CI −5.42, −1.06 mmHg; *low‐certainty evidence*) and diastolic BP (MD from baseline −3.07; 95% CI −4.88, −1.25 mmHg; *low‐certainty evidence*) (Figure 2, Table 2). In the post hoc sensitivity analyses, the overall effect sizes from the leave‐one‐out meta‐analyses are consistent, indicating that self‐care interventions may have lowered systolic and diastolic BP (Figure S5).

**TABLE 2:** Summary of Findings table (GRADE profile).

Outcomes	№ of participants (studies)	Certainty of the evidence (GRADE)	Relative effect (95% CI)	Anticipated absolute effects with no intervention, usual or standard care	
				Risk	Risk difference
Postpartum hypertension readmission assessed with: Incidence follow‐up: range 2 weeks to 9 months	605 (3 RCTs)	⨁⨁⨁◯ Moderate^a^	RR 0.35 (0.14 to 0.89)	126 per 1000	82 fewer per 1000 (109 fewer to 14 fewer)
Additional emergency department or office visit for hypertension not resulting in readmission assessed with: Incidence follow‐up: mean 15 days	403 (2 RCTs)	⨁⨁◯◯ Low^b^, ^c^	RR 2.22 (0.93 to 5.33)	34 per 1000	42 more per 1000 (2 fewer to 149 more)
Use of anti‐hypertensive medications assessed with: Incidence follow‐up: range 2 weeks to 12 months	1063 (7 RCTs)	⨁◯◯◯ Very low^c^, ^d^	RR 1.20 (0.82 to 1.75)	79 per 1000	16 more per 1000 (14 fewer to 59 more)
Systolic blood pressure (SBP) assessed with: mmHg follow‐up: range 3 months to 12 months	952 (9 RCTs)	⨁⨁◯◯ Low^e^, ^f^	—	—	MD 3.24 mmHg lower (5.42 lower to 1.06 lower)
Diastolic blood pressure (DBP) assessed with: mmHg follow‐up: range 3 months to 12 months	952 (9 RCTs)	⨁⨁◯◯ Low^e^, ^g^	—	—	MD 3.07 mmHg lower (4.88 lower to 1.25 lower)
Body mass index (BMI) assessed with: kg/m^2^ follow‐up: range 3 months to 12 months	503 (4 RCTs)	⨁◯◯◯ Very low^c^, ^h^	—	—	MD 0.46 kg/m2 lower (1.17 lower to 0.25 higher)
Smoking assessed with: Incidence follow‐up: range 9 months to 12 months	393 (2 RCTs)	⨁◯◯◯ Very low^c^, ^i^	RR 1.39 (0.54 to 3.55)	36 per 1000	14 more per 1000 (17 fewer to 93 more)
Physical activity assessed with: MET mins/week follow‐up: range 3 months to 9 months	175 (2 RCTs)	⨁◯◯◯ Very low^b^, ^c^	—	—	MD 388.55 MET mins/week lower (1931.75 lower to 1154.65 higher)
Total energy intake assessed with: Kcal/day	112 (2 RCTs)	⨁◯◯◯ Very low^b^, ^c^	—	—	MD 241.07 Kcal/day lower (720.59 lower to 238.45 higher)

*Note:* The risk in the intervention group (and its 95% confidence interval) is based on the assumed risk in the comparison group and the relative effect of the intervention (and its 95% CI). *GRADE Working Group grades of evidence*. High certainty: we are very confident that the true effect lies close to that of the estimate of the effect. Moderate certainty: we are moderately confident in the effect estimate: the true effect is likely to be close to the estimate of the effect, but there is a possibility that it is substantially different. Low certainty: our confidence in the effect estimate is limited: the true effect may be substantially different from the estimate of the effect. Very low certainty: we have very little confidence in the effect estimate: the true effect is likely to be substantially different from the estimate of effect.

Abbreviations: CI: confidence interval; MD: mean difference from baseline; RR: risk ratio.

^a^ Downgraded one level because of risk of bias: 2/3 studies at high risk of bias, mainly due to bias in the measurement of the outcome.

^b^ Downgraded one level because of risk of bias: both studies are at high risk of bias, mainly due to bias in the measurement of the outcome.

^c^ Downgraded one level: the confidence interval crosses no difference.

^d^ Downgraded two levels because of the risk of bias: 6/7 studies at high risk of bias with crucial limitations due to bias in missing outcome data (4/7 studies) and in the measurement of the outcome (2/7 studies).

^e^ Downgraded two levels because of risk of bias: 8/9 studies at high risk of bias. There is a high risk of bias due to a high loss of follow‐up (6/9 studies), lack of blinding in intervention implementation (3/9 studies), and potential selection of reported results (1/9 studies).

^f^ Did not downgrade due to inconsistency. I^2^ = 40.67% and was not explained by subgroup and sensitivity analyses; however, heterogeneity was primarily due to magnitude rather thaan n direction of effect.

^g^ Did not downgrade due to inconsistency. I^2^ = 50.91% and was not explained by subgroup and sensitivity analyses; however, heterogeneity was primarily due to magnitude rather than direction of effect.

^h^ Downgraded one level because of risk of bias: 3/4 studies were at a high risk of bias, with one of the studies having a potential selection bias of reported results.

^i^ Downgraded one level because of risk of bias: 1/2 studies were at a high risk of bias and a potential selection of reported results.

The effect of self‐care interventions on the risk of using antihypertensive medications is uncertain (RR 1.20; 95% CI 0.82, 1.75; 7 trials; 1063 women; *very low‐certainty evidence*) (Figure S3, Table 2). A non‐RCT reported no association between self‐care interventions and the risk of using anti‐hypertensive medications (adjusted RR 1.03; 95% CI 0.74, 1.44; *p* = 0.87) [56].

Self‐care interventions likely decrease the risk of postpartum hypertension readmission (RR 0.35; 95% CI 0.14, 0.89; 3 trials; 605 women; *moderate‐certainty evidence*) (Figure S4, Table 2). One non‐RCT (428 women) found that self‐care interventions reduced the risk of postpartum hypertension readmission (adjusted RR 0.12; 95% CI 0.01, 0.96; *p* = 0.045) and did not reduce the risk of hypertension‐related emergency or triage room visits (adjusted RR 0.81, 95% CI 0.36, 1.80; *p* = 0.81) [56].

Self‐care interventions may make no difference to the risk of additional emergency department or office visits for hypertension not resulting in readmission (RR 2.22; 95% CI 0.93, 5.33; 2 trials, 403 women; *low‐certainty evidence*) (Figure S4, Table 2).

We are uncertain whether self‐care interventions were associated with a decrease in body mass index (MD −0.46; 95% CI −1.17, 0.25 kg/m^2^; 4 trials; 503 women; *very low‐certainty evidence*), an increase in physical activity—the metabolic equivalent task (MET) (MD −528.67; 95% CI −502.53, 1559.87 MET mins/week; 2 trials; 170 women; *very low‐certainty evidence*), a decrease in total energy intake (MD −241.07; 95% CI −720.59, 238.45 Kcal/day; 2 trials; 112 women; *very low‐certainty evidence*), and a reduction in the risk of smoking (RR 1.39; 95% CI 0.5, 3.55; 2 trials; 393 women; *very low‐certainty evidence*) (Figure S4, Table 2).

### 3.7 Sub‐Group Analysis by Duration

There was strong evidence for no sub‐group effect (*p* = 0.12) on systolic BP and (*p* = 0.21) on diastolic BP, indicating that intervention duration did not modify the self‐care intervention effects compared to no intervention, usual care, or standard care. Nonetheless, the number of trials and participants who contributed data to the sub‐group short‐term was less than to the sub‐group medium‐term (4 trials vs. 5 trials), suggesting that these meta‐analyses could not detect subgroup differences (Figure 2).

A narrative summary of other secondary outcomes and detailed information on sensitivity analyses are described in Appendix S8.

## 4 Discussion

### 4.1 Main Findings

This review is the first to evaluate the effectiveness of self‐care interventions for reducing CVD risks and CVD outcomes in women with a history of HDP. Few of the sixteen studies involving 2200 women that met our eligibility criteria reported the same outcomes. For primary outcomes, there were insufficient studies to pool results for cardiovascular events and chronic hypertension. For secondary outcomes, we are uncertain whether self‐care interventions were associated with a reduction in the risks of using anti‐hypertensive medications, smoking, a decrease in BMI and total energy intake, and an increase in physical activity due to very low certainty of evidence. Additionally, self‐care interventions may make no difference to the risk of additional emergency department or office visits for hypertension not resulting in readmission due to low certainty of evidence. However, some self‐care interventions likely decrease the risk of postpartum hypertension readmission (moderate‐certainty evidence) and appear to be associated with a decrease in BP (low‐certainty evidence) compared to no intervention, usual, or standard care. Sensitivity analyses that replaced correlation values in studies with missing SD for change and leave‐one‐out meta‐analyses showed similar results to the primary analyses. Subgroup analyses showed that intervention duration did not modify the effect of self‐care interventions on BP compared to no intervention, usual care, or standard care. While the findings suggest some promise, further high‐quality studies with multiple and longer follow‐up periods are needed, and agreed‐upon core outcomes are reported.

### 4.2 Strengths and Limitations

This review has several strengths. It is the first meta‐analysis to evaluate the effectiveness of self‐care interventions for reducing the risk of CVDs in women with previous HDP. We assessed all RCTs for integrity and only included studies conducted with appropriate ethical standards to ensure quality, ethics, and benefits to research and society. We also assessed the certainty of the evidence for all pooled results, which was not done in the existing meta‐analysis [26], providing stronger evidence for the practice and research implications of the findings of our review.

There are some limitations in our review. Firstly, there is unexplained heterogeneity across review outcomes; however, exploring these was limited because few studies reported the same outcomes. There were too few studies for subgroup analyses of primary outcomes. Subgroup analyses by the duration of intervention were conducted for secondary outcomes; however, there was insufficient data for meta‐analysis by type of HDP experienced during pregnancy, postpartum period at enrolment, by type of interventions, by single or combined interventions, by reproductive age, and by settings. This unexplained heterogeneity and the high risk of bias complicated the interpretation of the findings of our review [59]. Secondly, studies included in this review mostly did not report on cardiovascular events—our primary outcome and—did not research various research settings, restricting the generalisability of our review's findings. Thirdly, all RCTs except one trial included in the meta‐analysis were judged to be at high risk of bias, limiting the certainty of evidence of this review. However, masking and self‐reported measures are commonly unavoidable, given the nature of self‐care interventions.

### 4.3 Interpretation

Existing literature highlights the need for longer‐term follow‐up and management to prevent CVDs in women with previous HDP. A 2024 rapid review of two studies involving 589 women with previous HDP in the United States and Canada reported that text‐based BP monitoring and antihypertensive therapy interventions up to 10 days were not associated with a reduction in BP, hospital readmission for hypertension, and persistence of hypertension [60]. The absence of long‐term follow‐up was considered to contribute to the lack of association [60]. Similarly, a 2021 integrative review of 194 women with previous HDP from two studies in the Netherlands reported little or no association between physical activity interventions and CVD risk reduction [61]. According to the authors, the trial durations (4 and 12 weeks) might have been too short to detect any change [61]. Both reviews highlighted a gap in the current literature on longer‐term follow‐up. Additionally, there is a persistent risk of developing CVDs for decades after the first occurrence of HDP [5, 6, 7], however, most women with a history of HDP might still have a low chance of absolute risk in a CVD risk assessment in the first 10 years [62]. In our review, pooled results from 2 RCTs that followed up with their participants in 6 and 12 months showed no clear benefit of self‐care intervention for lowering the incidence of the composite outcome of stage 1 or greater hypertension or on antihypertensive medications.

The current clinical recommendations are based on limited evidence for women with a history of HDP. The International Federation of Gynaecology and Obstetrics recommended that women with a history of HDP should adopt lifestyle modifications, including appropriate exercise and a heart‐healthy diet, to reduce CVD risks [62, 63]. However, except for one case–control and one cohort study on women with a history of HDP [64, 65], the evidence supporting their recommendations was not specific to this population. For example, recommendations for using a heart‐healthy diet to reduce the 10‐year Framingham Risk Score and exercise to lower CVD risks were based on meta‐analyses of adult men and women [66, 67]. Additionally, a 2019 review of international guidelines reported a lack of consensus in recommendations for frequency and length of follow‐up and an absence of referenced evidence on whether recommended interventions were effective for this population [27]. This lack of evidence and emphasis on consistent and long‐term management recommendations might explain why only one trial in our review followed participants for up to four years [50, 53]. Besides, findings from our review showed low to very low certainty of evidence despite the included trials incorporating recommended self‐care interventions in women with previous HDP. Furthermore, there were insufficient studies to explore the effectiveness of the types, single or combined interventions, timing, and settings of self‐care interventions, suggesting a significant knowledge gap in the prevention of CVDs in this high‐risk population.

Further trials and utilisation of the core outcome set of CVDs are needed. The American Heart Association—AHA (2017) emphasises that self‐care should not be overlooked in the effort to minimise CVD risks and outcomes [68]. Self‐care interventions could focus on the proposed mechanism of the underlying association between HDP and CVD risks, such as reducing inflammation [19]. One potential intervention that has not been explored in included trials is breastfeeding, which can lower CVD risks, as reported in a 2022 meta‐analysis in parous women [69]. Breastfeeding interventions can be implemented immediately after delivery, and women are only required to adhere to breastfeeding for up to 12 months postpartum compared to a lifelong commitment to maintaining a healthy lifestyle. This intervention may be particularly beneficial because women with a history of HDP often stop breastfeeding earlier than those without (−6.26, 95% CI −10.00, −2.51 weeks) in the first year postpartum [70]. Another potential intervention is to use biomarkers such as the calcitonin gene‐related peptide and adrenomedullin as an element in self‐care interventions [71, 72]. These biomarkers can identify individuals at an increased risk for developing CVDs [72], offering an alternative to the need for a long‐term follow‐up intervention. Furthermore, while the core outcome set of CVDs has been identified [32, 33, 34], it was not utilised in many of the included studies in this review. Using the core outcome set could improve the pooling of review outcomes and potentially improve heterogeneity.

It is essential to implement appropriate strategies to assess the effectiveness of self‐care interventions in this population. Effective interventions are critical in LMICs, where the burden of HDPs is highest. The incidence of HDP is highest in Africa, with a rate of 335 per 100 000 reproductive‐aged women in 2019, followed by Southeast Asia, with 136.8 per 100 000 and the Middle East, 121.4 per 100000 [73]. Nonetheless, studies included in this review were primarily conducted in high‐income countries, indicating a need for trials in LMICs. According to the WHO, self‐care interventions can be effective in diverse settings [16], and they could become even more essential in places with limited access to healthcare due to unavailability, inaccessibility, poor quality, and discrimination. For women, regardless of their postpartum period, maintaining self‐care practices can be challenging due to the demands of motherhood, efficacy, financial limitations, and adherence to social and cultural practices [74, 75]. Hence, future research should tailor self‐care interventions to the challenges and preferences of women with a history of HDP. For instance, a qualitative study reported that postpartum women preferred flexible, low‐intensity lifestyle intervention programs integrated into existing routine healthcare services [76]. Integrating self‐care interventions into the healthcare continuum allows healthcare professionals to provide early, continuous screening and consultation about the increased CVD risks [77, 78, 79], especially when self‐care interventions for this population are needed for further evaluation. Furthermore, this approach enables researchers to systematically collect data on women with previous HDP over time due to a persistent risk of developing CVDs [5, 6, 7]. Therefore, it is necessary for policymakers and public health practitioners to provide appropriate strategies to assess the viability of self‐care interventions, especially in LMICs.

## 5 Conclusion

This review investigated the effectiveness of self‐care interventions for the prevention of CVDs in women with previous HDP. Due to limited data on review outcomes, varying degrees of heterogeneity, and low to very low certainty of evidence, there is currently insufficient evidence for a recommendation of self‐care interventions for reducing the incidences of cardiovascular events, chronic hypertension, and reducing risk factors of CVDs in women with a history of HDP; although there was evidence that some self‐care interventions may lower blood pressure and likely decrease the risk of postpartum hypertension readmission. Larger trials with multiple and longer‐term follow‐ups, utilising the core outcome set of CVDs, may lead to different conclusions.

## Author Contributions

T.D.T.M., A.M. and M.M. are responsible for conceptualisation and methodology. T.D.T.M. and S.K. are responsible for formal analysis and data curation. A.M., J.V. and M.M. are responsible for supervision. P.‐Y.N. and L.R. are responsible for software. T.D.T.M. wrote the original draft of the manuscript. All authors were responsible for the review and editing of the manuscript.

## Ethics Statement

Ethical approval was not required as the study analysed publicly available data.

## Conflicts of Interest

The authors declare no conflicts of interest.

## Supporting information


Data S1.

## Data Availability

All data relevant to the study are included in the article or uploaded as Supporting Information.

## References

[1] L. A. Magee , M. A. Brown , D. R. Hall , et al., “The 2021 International Society for the Study of Hypertension in Pregnancy Classification, Diagnosis & Management Recommendations for International Practice,” Pregnancy Hypertens 27 (2022): 148–169. 35066406 10.1016/j.preghy.2021.09.008

[2] W. Wang , X. Xie , T. Yuan , et al., “Epidemiological Trends of Maternal Hypertensive Disorders of Pregnancy at the Global, Regional, and National Levels: A Population‐Based Study,” BMC Pregnancy and Childbirth 21, no. 1 (2021): 364. 33964896 10.1186/s12884-021-03809-2 PMC8106862

[3] L. Say , D. Chou , A. Gemmill , et al., “Global Causes of Maternal Death: A WHO Systematic Analysis,” Lancet Global Health 2, no. 6 (2014): e323–e333. 25103301 10.1016/S2214-109X(14)70227-X

[4] J. Sukmanee and T. Liabsuetrakul , “Risk of Future Cardiovascular Diseases in Different Years Postpartum After Hypertensive Disorders of Pregnancy: A Systematic Review and Meta‐Analysis,” Medicine (Baltimore) 101, no. 30 (2022): e29646. 35905265 10.1097/MD.0000000000029646 PMC9333537

[5] W. Ying , J. M. Catov , and P. Ouyang , “Hypertensive Disorders of Pregnancy and Future Maternal Cardiovascular Risk,” Journal of the American Heart Association 7, no. 17 (2018): e009382. 30371154 10.1161/JAHA.118.009382 PMC6201430

[6] P. Wu , R. Haththotuwa , C. S. Kwok , et al., “Preeclampsia and Future Cardiovascular Health,” Circulation. Cardiovascular Quality and Outcomes 10, no. 2 (2017): e003497, 10.1161/circoutcomes.116.003497. 28228456

[7] L. Bellamy , J. P. Casas , A. D. Hingorani , and D. J. Williams , “Pre‐Eclampsia and Risk of Cardiovascular Disease and Cancer in Later Life: Systematic Review and Meta‐Analysis,” BMJ 335, no. 7627 (2007): 974. 17975258 10.1136/bmj.39335.385301.BE PMC2072042

[8] N. M. Breetveld , C. Ghossein‐Doha , S. M. J. van Kuijk , et al., “Prevalence of Asymptomatic Heart Failure in Formerly Pre‐Eclamptic Women: A Cohort Study,” Ultrasound in Obstetrics & Gynecology 49, no. 1 (2017): 134–142. 27404208 10.1002/uog.16014

[9] A. S. Thayaparan , J. M. Said , S. A. Lowe , A. McLean , and Y. Yang , “Pre‐Eclampsia and Long‐Term Cardiac Dysfunction: A Review of Asymptomatic Cardiac Changes Existing Well Beyond the Post‐Partum Period,” Australasian Journal of Ultrasound in Medicine 22, no. 4 (2019): 234–244, 10.1002/ajum.12173. 34760564 PMC8411796

[10] K. G. Manton and K. Liu , “Projecting Chronic Disease Prevalence,” Medical Care 22, no. 6 (1984): 511–526, 10.1097/00005650-198406000-00002. 6738142

[11] World Health Organization , “Cardiovascular Diseases (CVDs),” 2021, https://www.who.int/news‐room/fact‐sheets/detail/cardiovascular‐diseases‐(cvds).

[12] K. Yeates , L. Lohfeld , J. Sleeth , F. Morales , Y. Rajkotia , and O. Ogedegbe , “A Global Perspective on Cardiovascular Disease in Vulnerable Populations,” Canadian Journal of Cardiology 31, no. 9 (2015): 1081–1093, 10.1016/j.cjca.2015.06.035. 26321432 PMC4787293

[13] British Heart Foundation the HI team , “Global Heart & Circulatory Diseases Factsheet,” 2024, https://www.bhf.org.uk/‐/media/files/for‐professionals/research/heart‐statistics/bhf‐cvd‐statistics‐global‐factsheet.pdf?rev=e61c05db17e9439a8c2e4720f6ca0a19&hash=6350DE1B2A19D939431D876311077C7B.British Heart Foundation.

[14] A. O. Mocumbi , “Cardiovascular Health Care in low‐ and Middle‐Income Countries,” Circulation 149, no. 8 (2024): 557–559. 38377254 10.1161/CIRCULATIONAHA.123.065717

[15] World Health Organization , “WHO Consolidated Guideline on Self‐Care Interventions for Health: Sexual and Reproductive Health and Rights,” 2019, http://www.ncbi.nlm.nih.gov/books/NBK544164/. 31334932

[16] World Health Organization , “Self‐Care Interventions for Health,” 2022, https://www.who.int/news‐room/fact‐sheets/detail/self‐care‐health‐interventions.

[17] International Self‐Care Foundation , “The Seven Pillars of Self‐Care,” https://isfglobal.org/practise‐self‐care/the‐seven‐pillars‐of‐self‐care/.

[18] B. Riegel , H. Westland , K. E. Freedland , et al., “Operational Definition of Self‐Care Interventions for Adults With Chronic Illness,” International Journal of Nursing Studies 129 (2022): 104231. 35344837 10.1016/j.ijnurstu.2022.104231

[19] C. S. Lee , N. C. Tkacs , and B. Riegel , “The Influence of Heart Failure Self‐Care on Health Outcomes: Hypothetical Cardioprotective Mechanisms,” Journal of Cardiovascular Nursing 24, no. 3 (2009): 179–189, 10.1097/JCN.0b013e31819b5419. 19279494 PMC2881684

[20] B. Hosseini , B. S. Berthon , A. Saedisomeolia , et al., “Effects of Fruit and Vegetable Consumption on Inflammatory Biomarkers and Immune Cell Populations: A Systematic Literature Review and Meta‐Analysis,” American Journal of Clinical Nutrition 108, no. 1 (2018): 136–155. 29931038 10.1093/ajcn/nqy082

[21] D. Aune , E. Giovannucci , P. Boffetta , et al., “Fruit and Vegetable Intake and the Risk of Cardiovascular Disease, Total Cancer and All‐Cause Mortality—A Systematic Review and Dose‐Response Meta‐Analysis of Prospective Studies,” International Journal of Epidemiology 46, no. 3 (2017): 1029–1056, 10.1093/ije/dyw319. 28338764 PMC5837313

[22] N. A. Lui , G. Jeyaram , and A. Henry , “Postpartum Interventions to Reduce Long‐Term Cardiovascular Disease Risk in Women After Hypertensive Disorders of Pregnancy: A Systematic Review,” Frontiers in Cardiovascular Medicine 6 (2019): 60, 10.3389/fcvm.2019.00160. 31803757 PMC6873287

[23] S. Behnam , N. Timmesfeld , and B. Arabin , “Lifestyle Interventions to Improve Pregnancy Outcomes: A Systematic Review and Specified Meta‐Analyses,” Geburtshilfe und Frauenheilkunde 82, no. 11 (2022): 1249–1264. 36339633 10.1055/a-1926-6636 PMC9634950

[24] P. T. Yeh , D. K. Rhee , C. E. Kennedy , et al., “Self‐Monitoring of Blood Pressure Among Women With Hypertensive Disorders of Pregnancy: A Systematic Review,” BMC Pregnancy and Childbirth 22, no. 1 (2022): 454. 35641913 10.1186/s12884-022-04751-7 PMC9152837

[25] M. G. Macphail , S. Juul , K. Wollny , et al., “Nutrition Interventions for Lowering Cardiovascular Risk After Hypertensive Disorders of Pregnancy: A Systematic Review,” CJC Open 6, no. 2, Part B (2024): 195–204. 38487049 10.1016/j.cjco.2023.10.018 PMC10935991

[26] M. Albadrani , M. Tobaiqi , and S. Al‐Dubai , “An Evaluation of the Efficacy and the Safety of Home Blood Pressure Monitoring in the Control of Hypertensive Disorders of Pregnancy in Both Pre and Postpartum Periods: A Systematic Review and Meta‐Analysis,” BMC Pregnancy and Childbirth 23, no. 1 (2023): 550. 37528352 10.1186/s12884-023-05663-w PMC10392017

[27] D. T. Gamble , B. Brikinns , P. K. Myint , and S. Bhattacharya , “Hypertensive Disorders of Pregnancy and Subsequent Cardiovascular Disease: Current National and International Guidelines and the Need for Future Research,” Frontiers in Cardiovascular Medicine 6 (2019): 55, 10.3389/fcvm.2019.00055. 31157237 PMC6533460

[28] M. J. Page , J. E. McKenzie , P. M. Bossuyt , et al., “The PRISMA 2020 Statement: An Updated Guideline for Reporting Systematic Reviews,” BMJ 372 (2021): n71. 33782057 10.1136/bmj.n71 PMC8005924

[29] The EndNote Team PCA , “EndNote 20 Windows and Macos: Release Notes,” 2023, https://support.clarivate.com/Endnote/s/article/EndNote‐20‐Release‐Notes?language=en_US.

[30] Veritas Health Innovation , “Covidence—Better Systematic Review Management,” https://www.covidence.org/.

[31] S. Weibel , M. Popp , S. Reis , N. Skoetz , P. Garner , and E. Sydenham , “Identifying and Managing Problematic Trials: A Research Integrity Assessment (RIA) Tool for Randomized Controlled Trials in Evidence Synthesis,” medRxiv 2022 (2022): 22275756v1, 10.1101/2022.05.31.22275756v1. PMC10551123 36054583

[32] A. Cowie , J. Buckley , P. Doherty , et al., “Standards and Core Components for Cardiovascular Disease Prevention and Rehabilitation,” Heart 105 (2019): 1–6. 30700518 10.1136/heartjnl-2018-314206 PMC6580752

[33] S. Woodruffe , L. Neubeck , R. A. Clark , et al., “Core Components of Cardiovascular Disease Secondary Prevention and Cardiac Rehabilitation 2014,” Heart, Lung & Circulation 24, no. 5 (2015): 430–441. 10.1016/j.hlc.2014.12.008 25637253

[34] J. Duffy , A. E. Cairns , D. Richards‐Doran , et al., “A Core Outcome Set for Pre‐Eclampsia Research: An International Consensus Development Study,” BJOG 127, no. 12 (2020): 1516–1526, 10.1111/1471-0528.16319. 32416644

[35] J. A. C. Sterne , J. Savović , M. J. Page , et al., “RoB 2: A Revised Tool for Assessing Risk of Bias in Randomised Trials,” BMJ 366 (2019): l4898. 31462531 10.1136/bmj.l4898

[36] J. A. Sterne , M. A. Hernán , B. C. Reeves , et al., “ROBINS‐I: A Tool for Assessing Risk of Bias in Non‐Randomised Studies of Interventions,” BMJ 355 (2016): i4919. 27733354 10.1136/bmj.i4919 PMC5062054

[37] L. A. McGuinness and J. P. T. Higgins , “Risk‐Of‐Bias VISualization (Robvis): An R Package and Shiny Web App for Visualizing Risk‐Of‐Bias Assessments,” Research Synthesis Methods 12, no. 1 (2021): 55–61, 10.1002/jrsm.1411. 32336025

[38] H. Schünemann , J. Brożek , G. Guyatt , and A. Oxman , GRADE Handbook Cochrane, (2013), https://gdt.gradepro.org/app/handbook/handbook.html.

[39] McMaster University and Evidence Prime , GRADEpro GDT: GRADEpro Guideline Development Tool [Software] GRADEpro, (2021), https://www.gradepro.org/.

[40] College Station S , “Stata Statistical Software: Release 16,” 2023, https://www.stata.com/stata‐news/news38‐2/.

[41] J. Higgins , J. Thomas , J. Chandler , et al., “Cochrane Handbook for Systematic Reviews of Interventions Version 6.4 (updated August 2023),” 2023, https://training.cochrane.org/handbook/current.

[42] H. E. C. Muijsers , P. Wu , O. W. H. van der Heijden , et al., “Home Blood Pressure Monitoring Detects Unrevealed Hypertension in Women With a History of Preeclampsia: Results of the BP‐PRESELF Study,” American Journal of Preventive Cardiology 12 (2022): 100429. 36425535 10.1016/j.ajpc.2022.100429 PMC9679579

[43] M. J. Hutchesson , R. Taylor , V. A. Shrewsbury , et al., “Be Healthe for Your Heart: A Pilot Randomized Controlled Trial Evaluating a Web‐Based Behavioral Intervention to Improve the Cardiovascular Health of Women With a History of Preeclampsia,” International Journal of Environmental Research and Public Health 17, no. 16 (2020): 5779. 32785044 10.3390/ijerph17165779 PMC7459885

[44] T. L. Wang , B. A. Quinn , R. Hart , et al., “The Effect of a Neonatal Sleep Intervention on Maternal Postpartum Hypertension: A Randomized Trial,” Am J Obstet Gynecol MFM 6, no. 2 (2024): 101239. 38072236 10.1016/j.ajogmf.2023.101239 PMC10922913

[45] J. Lewey , S. Murphy , D. Zhang , et al., “Effectiveness of a Text‐Based Gamification Intervention to Improve Physical Activity Among Postpartum Individuals With Hypertensive Disorders of Pregnancy: A Randomized Clinical Trial,” JAMA Cardiology 7, no. 6 (2022): 591–599. 35442393 10.1001/jamacardio.2022.0553 PMC9021982

[46] M. Riemer , S. Schulze , L. Wagner , et al., “Cardiovascular Risk Reduction in Women Following Hypertensive Disorders of Pregnancy – A Prospective, Randomised, Controlled Interventional Study,” Geburtshilfe und Frauenheilkunde 81, no. 8 (2021): 966–978, 10.1055/a-1345-8733. 34393260 PMC8354345

[47] M. Parfenova , A. M. Côté , A. Cumyn , et al., “Impact of an Educational Pamphlet on Knowledge About Health Risks After Hypertensive Disorders of Pregnancy: A Randomized Trial,” Journal of Obstetrics and Gynaecology Canada 43, no. 2 (2021): 182–190, 10.1016/j.jogc.2020.07.008. 33039316

[48] B. J. Arkerson , M. M. Finneran , S. R. Harris , et al., “Remote Monitoring Compared With in‐Office Surveillance of Blood Pressure in Patients With Pregnancy‐Related Hypertension,” Obstetrics and Gynecology 142, no. 4 (2023): 855–861. 37734091 10.1097/AOG.0000000000005327 PMC10510790

[49] A. Hauspurg , E. W. Seely , J. Rich‐Edwards , et al., “Postpartum Home Blood Pressure Monitoring and Lifestyle Intervention in Overweight and Obese Individuals the First Year After Gestational Hypertension or Pre‐Eclampsia: A Pilot Feasibility Trial,” BJOG 130, no. 7 (2023): 715–726, 10.1111/1471-0528.17381. 36655365 PMC10880812

[50] A. E. Cairns , K. L. Tucker , P. Leeson , et al., “Self‐Management of Postnatal Hypertension: The SNAP‐HT Trial,” Hypertension (0194911X) 72, no. 2 (2018): 425–432. 10.1161/HYPERTENSIONAHA.118.10911 29967037

[51] A. Hirshberg , K. Downes , and S. Srinivas , “Comparing Standard Office‐Based Follow‐Up With Text‐Based Remote Monitoring in the Management of Postpartum Hypertension: A Randomised Clinical Trial,” BMJ Quality and Safety 27, no. 11 (2018): 871–877, 10.1136/bmjqs-2018-007837. 29703800

[52] J. W. Rich‐Edwards , J. J. Stuart , G. Skurnik , et al., “Randomized Trial to Reduce Cardiovascular Risk in Women With Recent Preeclampsia,” Journal of Women's Health 28, no. 11 (2019): 1493–1504. 10.1089/jwh.2018.7523 31215837

[53] J. A. Kitt , R. L. Fox , A. E. Cairns , et al., “Short‐Term Postpartum Blood Pressure Self‐Management and Long‐Term Blood Pressure Control: A Randomized Controlled Trial,” Hypertension (0194911X) 78, no. 2 (2021): 469–479. 10.1161/HYPERTENSIONAHA.120.17101 PMC8260340 34176288

[54] J. Kitt , R. Fox , A. Frost , et al., “Long‐Term Blood Pressure Control After Hypertensive Pregnancy Following Physician‐Optimized Self‐Management: The POP‐HT Randomized Clinical Trial,” Journal of the American Medical Association 330, no. 20 (2023): 1991–1999. 37950919 10.1001/jama.2023.21523 PMC10640702

[55] J. M. Nicklas , L. Pyle , A. Soares , et al., “The Fit After Baby Randomized Controlled Trial: An mHealth Postpartum Lifestyle Intervention for Women With Elevated Cardiometabolic Risk,” PLoS One 19, no. 1 (2024): e0296244. 38194421 10.1371/journal.pone.0296244 PMC10775990

[56] K. K. Hoppe , N. Thomas , M. Zernick , et al., “Telehealth With Remote Blood Pressure Monitoring Compared With Standard Care for Postpartum Hypertension,” American Journal of Obstetrics and Gynecology 223, no. 4 (2020): 585–588. 32439388 10.1016/j.ajog.2020.05.027 PMC10428007

[57] E. Ekawati , S. Setyowati , and T. Budiati , “‘Sehati’ Health Education to Improve Physical and Psychological Adaptation of the Postpartum Women Having Pre‐Eclampsia,” Enfermería Clínica 29 (2019): 199–204.30975598

[58] J. A. Hutcheon , S. Lisonkova , and K. S. Joseph , “Epidemiology of Pre‐Eclampsia and the Other Hypertensive Disorders of Pregnancy,” Best Practice & Research. Clinical Obstetrics & Gynaecology 25, no. 4 (2011): 391–403. 21333604 10.1016/j.bpobgyn.2011.01.006

[59] P. B. Imrey , “Limitations of Meta‐Analyses of Studies With High Heterogeneity,” JAMA Network Open 3, no. 1 (2020): e1919325. 31922554 10.1001/jamanetworkopen.2019.19325

[60] D. Y. Quansah , R. Lewis , K. Savard , et al., “Cardiovascular Disease Risk Factor Interventions in Women With Prior Gestational Hypertensive Disorders or Diabetes in North America: A Rapid Review,” CJC Open 6, no. 2, Part B (2024): 153–164. 38487059 10.1016/j.cjco.2023.12.015 PMC10935679

[61] M. Byrnes and S. W. Buchholz , “Physical Activity and Cardiovascular Risk Factor Outcomes in Women With a History of Hypertensive Disorders of Pregnancy: Integrative Review,” Worldviews on Evidence‐Based Nursing 19, no. 1 (2022): 47–55, 10.1111/wvn.12537. 34482625

[62] L. C. Poon , L. Nguyen‐Hoang , G. N. Smith , et al., “Hypertensive Disorders of Pregnancy and Long‐Term Cardiovascular Health: FIGO Best Practice Advice,” International Journal of Gynecology & Obstetrics 160, no. S1 (2023): 22–34. 10.1002/ijgo.14540 36635079

[63] L. Nguyen‐Hoang , G. N. Smith , L. Bergman , et al., “FIGO Pregnancy Passport: A Useful Tool for Women and Their Healthcare Providers on Health Risks Following Pregnancy Complications,” International Journal of Gynecology & Obstetrics 162, no. 3 (2023): 787–791, 10.1002/ijgo.15029. 37485783

[64] R. R. Scholten , D. J. H. Thijssen , F. K. Lotgering , M. T. E. Hopman , and M. E. A. Spaanderman , “Cardiovascular Effects of Aerobic Exercise Training in Formerly Preeclamptic Women and Healthy Parous Control Subjects,” American Journal of Obstetrics and Gynecology 211, no. 5 (2014): 516. 10.1016/j.ajog.2014.04.025 24769012

[65] S. Timpka , J. J. Stuart , L. J. Tanz , E. B. Rimm , P. W. Franks , and J. W. Rich‐Edwards , “Lifestyle in Progression From Hypertensive Disorders of Pregnancy to Chronic Hypertension in Nurses' Health Study II: Observational Cohort Study,” BMJ 358 (2017): j3024. 28701338 10.1136/bmj.j3024 PMC5506852

[66] “Effects of the Dietary Approach to Stop Hypertension (DASH) Diet on Cardiovascular Risk Factors: A Systematic Review and Meta‐Analysis|British Journal of Nutrition|Cambridge Core,” 2024, https://www.cambridge.org/core/journals/british‐journal‐of‐nutrition/article/effects‐of‐the‐dietary‐approach‐to‐stop‐hypertension‐dash‐diet‐on‐cardiovascular‐risk‐factors‐a‐systematic‐review‐and‐metaanalysis/C3B37FC59A6FE257F3750C429C1251E6. 10.1017/S0007114514003341 25430608

[67] M. Nocon , T. Hiemann , F. Müller‐Riemenschneider , F. Thalau , S. Roll , and S. N. Willich , “Association of Physical Activity With All‐Cause and Cardiovascular Mortality: A Systematic Review and Meta‐Analysis,” European Journal of Cardiovascular Prevention and Rehabilitation 15, no. 3 (2008): 239–246, 10.1097/HJR.0b013e3282f55e09. 18525377

[68] B. Riegel , D. K. Moser , H. G. Buck , et al., “Self‐Care for the Prevention and Management of Cardiovascular Disease and Stroke,” Journal of the American Heart Association 6, no. 9 (2017): e006997. 28860232 10.1161/JAHA.117.006997 PMC5634314

[69] L. Tschiderer , L. Seekircher , S. K. Kunutsor , S. A. E. Peters , L. M. O'Keeffe , and P. Willeit , “Breastfeeding Is Associated With a Reduced Maternal Cardiovascular Risk: Systematic Review and Meta‐Analysis Involving Data From 8 Studies and 1 192 700 Parous Women,” Journal of the American Heart Association 11, no. 2 (2022): e022746. 35014854 10.1161/JAHA.121.022746 PMC9238515

[70] K. Horsley , K. Chaput , D. Da Costa , et al., “Hypertensive Disorders of Pregnancy and Breastfeeding Practices: A Secondary Analysis of Data From the all Our Families Cohort,” Acta Obstetricia et Gynecologica Scandinavica 101, no. 8 (2022): 871–879. 35610941 10.1111/aogs.14378 PMC9564688

[71] A. Shere , O. Eletta , and H. Goyal , “Circulating Blood Biomarkers in Essential Hypertension: A Literature Review,” Journal of Laboratory and Precision Medicine 2, no. 12 (2017): 3942.

[72] H. Mujadzic , J. Skeete , and D. J. DiPette , “Historical, Present, and Emerging Biomarkers in Hypertension: A Narrative Review,” Journal of Laboratory and Precision Medicine 7 (2022): 7407.

[73] L. Jiang , K. Tang , L. A. Magee , et al., “A Global View of Hypertensive Disorders and Diabetes Mellitus During Pregnancy,” Nature Reviews. Endocrinology 18, no. 12 (2022): 760–775, 10.1038/s41574-022-00734-y. PMC9483536 36109676

[74] J. L. Barkin and K. L. Wisner , “The Role of Maternal Self‐Care in New Motherhood,” Midwifery 29, no. 9 (2013): 1050–1055. 23415369 10.1016/j.midw.2012.10.001 PMC7081756

[75] E. Sacks , K. Finlayson , V. Brizuela , et al., “Factors That Influence Uptake of Routine Postnatal Care: Findings on Women's Perspectives From a Qualitative Evidence Synthesis,” PLoS One 17, no. 8 (2022): e0270264. 35960752 10.1371/journal.pone.0270264 PMC9374256

[76] M. Makama , M. Chen , L. J. Moran , et al., “Postpartum Women's Preferences for Lifestyle Intervention After Childbirth: A Multi‐Methods Study Using the TIDieR Checklist,” Nutrients 14, no. 20 (2022): 4229. 36296913 10.3390/nu14204229 PMC9611337

[77] C. X. Xie , L. Sun , E. Ingram , et al., “Use of Routine Healthcare Data in Randomised Implementation Trials: A Methodological Mixed‐Methods Systematic Review,” Implementation Science 18 (2023): 47. 37784099 10.1186/s13012-023-01300-4 PMC10544368

[78] S. Marschner , A. Pant , A. Henry , et al., “Cardiovascular Risk Management Following Gestational Diabetes and Hypertensive Disorders of Pregnancy: A Narrative Review,” Medical Journal of Australia 2023 (2023): 1. 10.5694/mja2.51932 PMC10953444 37149790

[79] D. P. French , L. M. Miles , D. Elbourne , et al., “Reducing Bias in Trials due to Reactions to Measurement: Experts Produced Recommendations Informed by Evidence,” Journal of Clinical Epidemiology 139 (2021): 130–139. 34229092 10.1016/j.jclinepi.2021.06.028 PMC7614249

