# Supplementary material for: Self‐Care Interventions for Preventing Cardiovascular Diseases After Hypertensive Pregnancy Disorders: A Systematic Review and Meta‐Analysis
Source: BJOG. 2025 Mar 28;132(10):1350–61. doi: 10.1111/1471-0528.18152 (PMC12315088; doi:10.1111/1471-0528.18152)
Supplement: Supplementary file 1 — Data S1. [file BJO-132-1350-s001.docx]

**Supplemental Materials**

Contents

[Appendix S1_the Preferred Reporting Items for Systematic Reviews and Meta-Analyses 2020 (PRISMA) Checklist 2](#_Toc189509279)

[Appendix S2_Search strategy 4](#_Toc189509280)

[Appendix S3_List of secondary outcomes 19](#_Toc189509281)

[Appendix S4_Supplementary Methods 20](#_Toc189509282)

[Appendix S5_Included studies and relevant studies 23](#_Toc189509283)

[Appendix S6_Additional information on characteristics of included studies 38](#_Toc189509284)

[Appendix S7_Risk of bias assessments 42](#_Toc189509285)

[Appendix S8_Secondary outcomes, sub-group analyses, sensitivity analyses and post-hoc analyses 50](#_Toc189509287)

[Appendix S9_Protocol 72](#_Toc189509288)

**Appendix S1_The Preferred Reporting Items for Systematic Reviews and Meta-Analyses 2020 (PRISMA) Checklist**

**Table S1_The PRISMA Checklist**

| **Section and Topic** | **Item #** | **Checklist item** | **Location where item is reported** |
| --- | --- | --- | --- |
| **TITLE** | | |  |
| Title | 1 | Identify the report as a systematic review. | 1 |
| **ABSTRACT** | | |  |
| Abstract | 2 | See the PRISMA 2020 for Abstracts checklist. | 1-2 |
| **INTRODUCTION** | | |  |
| Rationale | 3 | Describe the rationale for the review in the context of existing knowledge. | 3-4 |
| Objectives | 4 | Provide an explicit statement of the objective(s) or question(s) the review addresses. | 4 |
| **METHODS** | | |  |
| Eligibility criteria | 5 | Specify the inclusion and exclusion criteria for the review and how studies were grouped for the syntheses. | 4-5 |
| Information sources | 6 | Specify all databases, registers, websites, organisations, reference lists and other sources searched or consulted to identify studies. Specify the date when each source was last searched or consulted. | 5 |
| Search strategy | 7 | Present the full search strategies for all databases, registers and websites, including any filters and limits used. | Appendix S2 |
| Selection process | 8 | Specify the methods used to decide whether a study met the inclusion criteria of the review, including how many reviewers screened each record and each report retrieved, whether they worked independently, and if applicable, details of automation tools used in the process. | 5-6 |
| Data collection process | 9 | Specify the methods used to collect data from reports, including how many reviewers collected data from each report, whether they worked independently, any processes for obtaining or confirming data from study investigators, and if applicable, details of automation tools used in the process. | 5-6 |
| Data items | 10a | List and define all outcomes for which data were sought. Specify whether all results that were compatible with each outcome domain in each study were sought (e.g. for all measures, time points, analyses), and if not, the methods used to decide which results to collect. | 6 and Appendix S3 |
|  | 10b | List and define all other variables for which data were sought (e.g. participant and intervention characteristics, funding sources). Describe any assumptions made about any missing or unclear information. | 6 |
| Study risk of bias assessment | 11 | Specify the methods used to assess risk of bias in the included studies, including details of the tool(s) used, how many reviewers assessed each study and whether they worked independently, and if applicable, details of automation tools used in the process. | 7 |
| Effect measures | 12 | Specify for each outcome the effect measure(s) (e.g. risk ratio, mean difference) used in the synthesis or presentation of results. | 7 |
| Synthesis methods | 13a | Describe the processes used to decide which studies were eligible for each synthesis (e.g. tabulating the study intervention characteristics and comparing against the planned groups for each synthesis (item #5)). | 7 |
|  | 13b | Describe any methods required to prepare the data for presentation or synthesis, such as handling of missing summary statistics, or data conversions. | 7 and Appendix S4 |
|  | 13c | Describe any methods used to tabulate or visually display results of individual studies and syntheses. | 7 and Appendix S4 |
|  | 13d | Describe any methods used to synthesize results and provide a rationale for the choice(s). If meta-analysis was performed, describe the model(s), method(s) to identify the presence and extent of statistical heterogeneity, and software package(s) used. | 7 and Appendix S4 |
|  | 13e | Describe any methods used to explore possible causes of heterogeneity among study results (e.g. subgroup analysis, meta-regression). | 7 and Appendix S4 |
|  | 13f | Describe any sensitivity analyses conducted to assess robustness of the synthesized results. | 7 and Appendix S4 |
| Reporting bias assessment | 14 | Describe any methods used to assess risk of bias due to missing results in a synthesis (arising from reporting biases). | 7 and Appendix S4 |
| Certainty assessment | 15 | Describe any methods used to assess certainty (or confidence) in the body of evidence for an outcome. | 7 and Appendix S4 |
| **RESULTS** | | |  |
| Study selection | 16a | Describe the results of the search and selection process, from the number of records identified in the search to the number of studies included in the review, ideally using a flow diagram. | 8 |
|  | 16b | Cite studies that might appear to meet the inclusion criteria, but which were excluded, and explain why they were excluded. | Appendix S5 |
| Study characteristics | 17 | Cite each included study and present its characteristics. | 8-9 and Table 1 and Appendix S6 |
| Risk of bias in studies | 18 | Present assessments of risk of bias for each included study. | 9 and Appendix S7 |
| Results of individual studies | 19 | For all outcomes, present, for each study: (a) summary statistics for each group (where appropriate) and (b) an effect estimate and its precision (e.g. confidence/credible interval), ideally using structured tables or plots. | 9-11 and Table 2 |
| Results of syntheses | 20a | For each synthesis, briefly summarise the characteristics and risk of bias among contributing studies. | 9-11 |
|  | 20b | Present results of all statistical syntheses conducted. If meta-analysis was done, present for each the summary estimate and its precision (e.g. confidence/credible interval) and measures of statistical heterogeneity. If comparing groups, describe the direction of the effect. | 9-11 and Table 2 |
|  | 20c | Present results of all investigations of possible causes of heterogeneity among study results. | Appendix S8 |
|  | 20d | Present results of all sensitivity analyses conducted to assess the robustness of the synthesized results. | 10 and Appendix S8 |
| Reporting biases | 21 | Present assessments of risk of bias due to missing results (arising from reporting biases) for each synthesis assessed. | Appendix S7 |
| Certainty of evidence | 22 | Present assessments of certainty (or confidence) in the body of evidence for each outcome assessed. | Table 2 |
| **DISCUSSION** | | |  |
| Discussion | 23a | Provide a general interpretation of the results in the context of other evidence. | 12 |
|  | 23b | Discuss any limitations of the evidence included in the review. | 12-13 |
|  | 23c | Discuss any limitations of the review processes used. | 13 |
|  | 23d | Discuss implications of the results for practice, policy, and future research. | 13-16 |
| **OTHER INFORMATION** | | |  |
| Registration and protocol | 24a | Provide registration information for the review, including register name and registration number, or state that the review was not registered. | 4 |
|  | 24b | Indicate where the review protocol can be accessed, or state that a protocol was not prepared. | 4 and Appendix S9 |
|  | 24c | Describe and explain any amendments to information provided at registration or in the protocol. | NA |
| Support | 25 | Describe sources of financial or non-financial support for the review, and the role of the funders or sponsors in the review. | 29 |
| Competing interests | 26 | Declare any competing interests of review authors. | 28 |
| Availability of data, code and other materials | 27 | Report which of the following are publicly available and where they can be found: template data collection forms; data extracted from included studies; data used for all analyses; analytic code; any other materials used in the review. | 4 |

**Appendix S2_Search strategy**

**Table S2. Concept A – Hypertensive disorder in pregnancy**

| **CINAHL subject thesaurus** | (MH "Eclampsia") OR (MH "Pre-Eclampsia") OR (MH "Pregnancy-Induced Hypertension") OR (MH "HELLP Syndrome") |
| --- | --- |
| **Medline (MeSH)** | hypertension, pregnancy-induced/ or eclampsia/ or hellp syndrome/ or pre-eclampsia/  or  (Pregnancy Complications/ or Pregnancy/) and Hypertension/ |
| **EMBASE (MeSH)** | maternal hypertension/ or pregnancy complication/ or hellp syndrome/  or  preeclampsia/ or "eclampsia and preeclampsia"/  or  (Pregnancy Complications/ or Pregnancy/) and Hypertension/ |
| **PsycINFO(MeSH)** | (obstetrical complications/ or Pregnancy/) and Hypertension/  or  (blood pressure/ or blood pressure disorders/) and pregnancy/  or  preeclampsia/ |
| **Cochrane(MeSH)** | [Hypertension, Pregnancy-Induced] this term only  or  [Eclampsia] this term only  or  [Pre-Eclampsia] this term only |
| **Textwords (CINAHL)** | (pregnan* or maternal or gestational) N3 (hypertens* or high blood pressure) |
| **Text words (Medline/EMBASE/PsycINFO)** | ((pregnan* or maternal or gestational) adj3 (hypertens* or high blood pressure)).mp.  or  (preeclampsia or pre-eclampsia or eclampsia).mp. |
| **Text words (Cochrane)** | ((pregnan* or maternal or gestational) NEAR/3 (hypertens* or high blood pressure)):ti,ab,kw |

**Table S3. Concept B – Self-care**

| **CINAHL subject thesaurus** | (MH "Self Assessment") OR (MH "Self-Diagnosis+") OR (MH "Self Report+") OR (MH "Self Administration+") OR (MH "Self Regulation+") OR (MH "Self-Management") OR (MH "Self Medication") OR (MH "Self Care Agency") OR (MH "Self Care+") OR (MH "Self-Testing") OR (MH "Self-Efficacy") OR (MH "Self-Awareness") OR (MH "Patient Self Determination Act") OR (MH "Patient Autonomy")  or  (MH "Counseling+") OR (MH "Nutritional Counseling") OR (MH "Peer Counseling") OR (MH "Mental Health Counseling")  or  ( (MH "Diet") OR (MH "Diet, Reducing") ) OR ( (MH "Diet Therapy") OR (MH "DASH Diet") OR (MH "Diet, Fat-Restricted") OR (MH "Diet, Low Carbohydrate") )  or  (MH "Home Nutritional Support") OR (MH "Dietary Supplementation") OR (MH "Nutritional Support")  or  (MH "Exercise") OR (MH "Therapeutic Exercise")  or  (MH "Weight Control") OR (MH "Weight Reduction Programs")  or  (MH "Health Education") OR (MH "Diabetes Education") OR (MH "Nutrition Education") OR (MH "Patient Education")  or  (MH "Consumer Health Information") OR MH "Home Health Care Information Systems"  or  (MH "Health Literacy")  or  (MH "Breast Feeding Promotion") OR (MH "Health Promotion")  or  ( (MH "Health Behavior") OR (MH "Patient Compliance") OR (MH "Medication Compliance") ) OR ( (MH "Risk Taking Behavior") OR (MH "Help Seeking Behavior") OR (MH "Behavioral Changes") OR (MH "Maternal Behavior") ) OR ( (MH "Behavior Modification") OR (MH "Behavior Therapy") )  or  (MH "Sleep Hygiene+")  or  (MH "Smoking Cessation") OR (MH "Smoking Cessation Programs") OR (MH "Substance Abstinence") OR (MH "Tobacco Use Cessation Products")  or  (MH "Harm Reduction")  or  (MH "Alcohol Abstinence") OR (MH "Drinking Behavior") OR (MH "Alcohol Drinking")  or  (MH "Psychotherapy")  or  (MH "Life Style Changes") OR (MH "Life Style+") OR (MH "Work-Life Balance")  or  (MH "Breast Feeding") |
| --- | --- |
| **Medline (MeSH)** | self care/ or self administration/ or self medication/ or self-testing/  or  counseling/ or directive counseling/ or motivational interviewing/ or distance counseling/  or  Diet/ or Nutrition Therapy/ or diet therapy/ or caloric restriction/ or diet, carbohydrate-restricted/ or diet, fat-restricted/ or diet, mediterranean/ or diet, reducing/ or dietary approaches to stop hypertension/ or nutritional support/ or diet, healthy/  Or  Exercise/ or Exercise Therapy/ or Weight Loss/  or  health education/ or consumer health information/ or health literacy/ or health education, dental/ or health promotion/ or healthy people programs/ or weight reduction programs/ or patient education as topic/ or smoking prevention/ or "physical education and training"/  or  health behavior/ or health risk behaviors/ or self-examination/ or sleep hygiene/ or smoking cessation/ or smoking reduction/ or "tobacco use cessation"/ or "treatment adherence and compliance"/ or patient compliance/ or medication adherence/  or  obesity management/  or  drinking behavior/ or alcohol abstinence/ or alcohol drinking/  or  psychotherapy/ or behavior therapy/  or  life style/ or healthy lifestyle/  or breast feeding/ or breast milk expression/ |
| **EMBASE (MeSH)** | self care/ or self care agency/ or self care education/ or self help/ or self medication/ or self-testing/  or  self-care software/ or mobile health application/  or  drug self administration/  or  patient monitoring/ or home monitoring/ or self monitoring/ or telemonitoring/ or home care/ or home mental health care/  or  blood pressure monitoring/ or blood pressure measurement/  or  counseling/ or directive counseling/ or e-counseling/ or motivational interviewing/ or nutritional counseling/ or patient counseling/ or patient guidance/ or peer counseling/ or psychological counseling/  or  diet/ or diet therapy/ or healthy diet/ or low carbohydrate diet/ or dash diet/ or high fiber diet/ or low calorie diet/ or low fat diet/ or nutritional support/ or diet restriction/ or caloric restriction/  or  exercise/ or physical activity/  or  body weight management/ or body weight maintenance/ or obesity management/ or weight cutting/ or weight loss program/  or  health education/ or breast feeding education/ or health literacy/ or health promotion/ or nutrition education/ or patient education/ or psychoeducation/ or self care education/ or sleep education/  or  health behavior/ or alcohol abstinence/ or attitude to health/ or behavioral risk factor surveillance system/ or drinking behavior/ or harm reduction/ or health belief/ or health belief model/ or high risk behavior/ or risk reduction/ or sleep hygiene/ or smoking cessation/ or smoking reduction/  or  psychotherapy/  or  "lifestyle and related phenomena"/ or healthy lifestyle/ or lifestyle modification/ or work-life balance/  or  medication adherence monitoring system/  or  patient compliance/ or dietary compliance/ or medication compliance/  or  breast feeding/ or breast milk expression/ |
| **PsycINFO (MeSH)** | health behavior/ or health risk behavior/ or healthy eating/ or preventive health behavior/ or self-care/  or  drug self administration/  or  self-care skills/  or  self-management/ or self-help techniques/ or self-instructional training/ or self-determination/ or self-evaluation/ or self-monitoring/ or self-regulation/ or self-reinforcement/  or  health care seeking behavior/ or self-referral/  or  counseling/ or community counseling/ or educational counseling/ or group counseling/ or peer counseling/ or psychotherapeutic counseling/  or  diets/ or dietary treatment/  or  nutrition/ or alcoholic beverages/ or "beverages (nonalcoholic)"/ or carbohydrates/ or dietary supplements/ or mealtimes/  or  physical activity/ or exercise/  or  physical therapy/ or exercise therapy/  or  health education/ or drug education/ or mental health education/ or public health campaigns/  or  health information/ or digital health resources/  or  health literacy/ or mental health literacy/  or  health promotion/  or  smoking prevention/ or "substance use prevention"/  or  smoking cessation/ or tobacco control/ or tobacco smoking/  or  treatment compliance/  or  drinking behavior/  or  sobriety/  or  alcohols/  or  psychotherapy/  or  behavior therapy/ or behavior modification/ or cognitive behavior therapy/ or relaxation therapy/  or  lifestyle/ or lifestyle changes/  or  breast feeding/  or  weight loss/ or weight control/ |
| **Cochrane (MeSH)** | [Self Care] this term only  or  [Self Administration] this term only  or  [Self Medication] this term only  or  [Self-Testing] this term only  or  [Counseling] this term only  or  [Directive Counseling] this term only  or  [Motivational Interviewing] this term only  or  [Distance Counseling] this term only  or  [Diet] this term only  or  [Diet Therapy] this term only  or  [Caloric Restriction] this term only  or  [Nutrition Therapy] this term only  or  [Diet, Carbohydrate-Restricted] this term only  or  [Diet, Fat-Restricted] this term only  or  [Diet, Reducing] this term only  or  [Exercise] this term only  or  [Dietary Approaches To Stop Hypertension] this term only  or  [Nutritional Support] this term only  or  [Diet, Healthy] this term only  or  [Obesity Management] this term only  or  [Exercise Therapy] this term only  or  [Consumer Health Information] this term only  or  [Health Literacy] this term only  or  [Health Promotion] this term only  or  [Healthy People Programs] this term only  or  [Consumer Health Information] this term only  or  [Health Behavior] explode all trees  or  [Weight Reduction Programs] this term only  or  [Weight Loss] this term only  or  [Patient Education as Topic] this term only  or  [Smoking Prevention] this term only  or  : [Smoking Cessation] this term only  or  [Smoking Reduction] this term only  or  [Tobacco Use Cessation] this term only  or  [Physical Education and Training] this term only  or  [Obesity Management] this term only  or  : [Health Risk Behaviors] this term only  or  [Self-Examination] this term only  or  [Sleep Hygiene] this term only  or  [Treatment Adherence and Compliance] this term only  or  [Patient Compliance] this term only  or  [Medication Adherence] this term only  or  [Drinking Behavior] this term only  or  [Alcohol Abstinence] this term only  or  [Alcohol Drinking] this term only  or  [Psychotherapy] this term only  or  [Behavior Therapy] this term only  or  [Life Style] explode all trees  or  [Healthy Lifestyle] this term only  or  [Breast Milk Expression] this term only  or  [Breast Feeding] this term only |
| **Textwords (CINAHL)** | “self-car*” or “selfcar*” or “self-manag*” or “selfmanag*” or “self-medica*” or “selfmedica*” or “self-treat*” or “selftreat*” or “self-examin*” or “selfexamin*” or “self-injectio*” or “selfinjectio*” or “self-administr*” or “selfadministr*” or “self-use*” or “selfuse*” or “self-test*” or “selftest*” or “self-sampl*” or “selfsampl*” or “self-screen*” or “selfscreen*” or “self-diagnos*” or “selfdiagnos*” or “self-collect*” or “selfcollect*” or “self-monitor*” or “selfmonitor*” or “self-awar*” or “selfawar*” or “self-help*” or “selfhelp*” or “self-educat*” or “selfeducat*” or “self-regulat*” or “selfregulat*” or “self-efficac*” or “selfefficac*” or “self-determin*” or “self-determin*” or “self-care activit*” or “selfcare activi*” or “self-nurtur*” or “selfnurtur*” or “self-evaluat*” or “selfevaluat*” or  ((alcoho* or drink*) N2 (quit* or cessat* or stop* or prevent* or abstinen* or reduc* or interven* or treat* or manag* or improv* or contro* or loss*)) or  counsel?* or motivat* or (“patient-orient*” N2 care)  or  exercis* or physical activit* or  (health* N2 (educat* or inform* or promot* or people program* or literacy*)) or  ((obes* or overweigh* or weigh*) N2 (interven* or treat* or manag* or improv* or contro* or reduc* or loss* or losin*)) or  ((nutrition* or diet*) N2 (therap* or treatmen* or approac* or suppor*)) or ((health* or mediterranea* or dash* or diabet* or “low carbohydrat*” or “carbohydrat* restrict*” or “high fibe*” or “low calori*” or “calori* restrict*” or “low fat*” or “fat restrict*” or “low glycem* inde*”) N1 diet*) or ((calori* or diet*) N2 restrict*))  or  ((smok* or tobacc* or cigarett*) N2 (quit* or cessat* or stop* or prevent* or abstinen* or reduc*))  or  behavio#r* or psychotherap* or “psycho-therap*” or “psycholo* therap*” or “psycholo* treat*” or “psycholo* interven*”  or  adher* or complian* or comply* or complie*  or  lifestyl* or "life styl*  or  breast feed* or breastfeed* or breast milk* or breastmilk* or breast fed or  or  coping* |
| **Text words (Medline/EMBASE/ PsycINFO)** | (self-car* or selfcar* or self-manag* or selfmanag* or self-medica* or selfmedica* or self-treat* or selftreat* or self-examin* or selfexamin* or self-injectio* or selfinjectio* or self-administr* or selfadministr* or self-use* or selfuse* or self-test* or selftest* or self-sampl* or selfsampl* or self-screen* or selfscreen* or self-diagnos* or selfdiagnos* or self-collect* or selfcollect* or self-monitor* or selfmonitor* or self-awar* or selfawar* or self-help* or selfhelp* or self-educat* or selfeducat* or self-regulat* or selfregulat* or self-efficac* or selfefficac* or self-determin* or self-determin* or self-care activit* or selfcare activi* or self-nurtur* or selfnurtur* or self-evaluat* or selfevaluat*).mp.  or  (counsel?* or motivat*).mp.  or  ((obes* or overweigh* or weigh*) adj3 (interven* or treat* or manag* or improv* or contro* or reduc* or losing* or lose* or lost* or loss*)).mp.  or  (blood pressure* adj3 (interven* or treat* or manag* or improv* or contro*)).mp.  or  (health* adj3 (educat* or inform* or promot* or people program* or literacy*)).mp.  or  (exercis* or physical activit*).mp  or  (((nutrition* or diet*) adj3 (therap* or treatmen* or approac* or suppor*)) or ((health* or mediterranea* or dash* or diabet* or low carbohydrat* or carbohydrat* restrict* or high fibe* or low calori* or calori* restrict* or low fat* or fat restrict* or low glycem* inde*) adj2 diet*) or ((calori* or diet*) adj3 restrict*)).mp.  or  ((behavio?r* adj (interven* or treat* or manag* or improv* or approac* or modifi*)) or (psychotherap* or (psycho* adj (therap* or treat* or interven*))) or (health* behavio?r* or drink* behavio?r* or sedentary behavio?r* or risk behavio?r* or behavio?r* risk)).mp.  or  (lifestyl* or life styl*).mp.  or  ((smok* or tobacc* or cigarett*) adj3 (quit* or cessat* or stop* or prevent* or abstinen* or reduc*)).mp.  or  ((alcoho* or drink*) adj3 (quit* or cessat* or stop* or prevent* or abstinen* or reduc* or interven* or treat* or manag* or improv* or contro* or loss*)).mp.  or  (adher* or complian* or comply* or complie*).mp.  or  coping*.mp.  or  (breast feed* or breastfeed* or breast milk* or breastmilk* or breast fed* or breastfed*).mp. |
| **Text words (Cochrane)** | (counsel* or motivat*):ti,ab,kw  or  (self-car* or selfcar* or self-manag* or selfmanag* or self-medica* or selfmedica* or self-treat* or selftreat* or self-examin* or selfexamin* or self-injectio* or selfinjectio* or self-administr* or selfadministr* or self-use* or selfuse* or self-test* or selftest* or self-sampl* or selfsampl* or self-screen* or selfscreen* or self-diagnos* or selfdiagnos* or self-collect* or selfcollect* or self-monitor* or selfmonitor* or self-awar* or selfawar* or self-help* or selfhelp* or self-educat* or selfeducat* or self-regulat* or selfregulat* or self-efficac* or selfefficac* or self-determin* or self-determin* or self-care activit* or selfcare activi* or self-nurtur* or selfnurtur* or self-evaluat* or selfevaluat*):ti,ab,kw  or  (exercis* or physical activit*):ti,ab,kw  or  (educat* or (health* NEAR/2 inform*) or litera* or healt* promot* or healthy people progra*):ti,ab,kw  or  (((obes* or overweigh* or weigh*) NEAR/2 (interven* or treat* or manag* or improv* or contro* or reduc* or loss* or losin*))):ti,ab,kw  or  ((smok* or tobacc* or cigarett*) NEAR/2 (quit* or cessat* or stop* or prevent* or abstinen* or reduc*)):ti,ab,kw  or  ((((nutrition* or diet*) NEAR/2 (therap* or treatmen* or approac* or suppor*)) or ((health* or dash* or diabet* or low carbohydrat* or carbohydrat* restrict* or high fibe* or low calori* or calori* restrict* or low fat* or fat restrict*) NEAR/2 diet*) or ((calori* or diet*) NEAR/2 restrict*))):ti,ab,kw  or  ((behavi*r* NEAR/0 (interven* or treat* or manag* or improv* or approac* or modifi*)) or (psychotherap* or (psycho* adj (therap* or treat* or interven*))) or (health* behavi*r* or drink* behavi*r* or sedentary behavi*r* or risk behavi*r* or behavi*r* risk)):ti,ab,kw  or  ((alcoho* or drink*) NEAR/2 (quit* or cessat* or stop* or prevent* or abstinen* or reduc* or interven* or treat* or manag* or improv* or contro* or loss*)):ti,ab,kw  or  (adher* or complian* or comply* or complie*):ti,ab,kw  or  (lifestyl* or life styl*):ti,ab,kw  or  (coping*):ti,ab,kw  or  ((breast feed* or breastfeed* or breast milk* or breastmilk* or breast fed* or breastfed*)):ti,ab,kw |

**Appendix S3_List of secondary outcomes**

- - Modifiable
- BMI
- Diabetes/Impaired glucose tolerance
- Blood pressure (mean diastolic and systolic blood pressure)
- Lipid levels
- Physical activity, as measured by the study
- Dietary intake as measured by the study
- Breastfeeding status (e.g., self-reported)
- Psychological status (Health-related quality of life, depression and anxiety assessed using a validated score)
- Smoking status (e.g., self-reported, salivary cotinine)
- Caffeine use status
- Alcohol use status (e.g., self-reported, referral to alcohol rehab)
- Illicit drug use (e.g., self-reported, referral to drug rehab)
- Social harms (e.g., stigma, intimate partner violence
- Autonomy (e.g., self-efficacy, empowerment)
- Antihypertensive medication requirement
- Other as defined by the study
  - Compliance of patients or participant retention with long-term follow-up, as measured by attendance records.

**Appendix S4_Supplementary Methods**

*Additional study inclusion criteria*

Reviews, meta-analyses, observational cohort studies, cross-sectional studies, case series, case studies, case reports, dissertations and theses, reports published as abstracts only, conference abstracts, and qualitative studies were not eligible. Research letters reporting on trials were included if they provided sufficient information for data extraction.

*Dealing with the missing standard deviation*

For studies with missing standard deviation (SD) for changes from baseline, we calculated the missing SDs using the formula recommended in Chapter 6, Cochrane Handbook,^1^ with a correlation coefficient conservatively set at 0.5 as previously reported.^2^


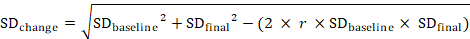


*Unit of analysis issues*

For the study with multiple intervention arms, if data were reported in mean and SD, we combined those arms to create a single pair-wise comparison, as recommended in Chapter 6, Cochrane Handbook.^1^ If the study reported data in median, we presented narratively review outcomes in each intervention arm.


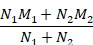

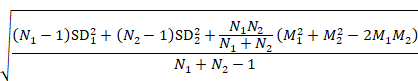

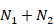


Combined sample size

Combined mean

Combined standard deviation

*Sensitivity analysis*

Two sensitivity analyses were conducted. Results of sensitivity analyses were tabulated as recommended in Chapter 10, Cochrane Handbook.^1^

- For outcomes involving the imputed SDs for changes from baseline, we conducted meta-analyses to evaluate the impact of changing the correlation coefficients from r=0.5 to r=0.7. The assumption of using a correlation coefficient of 0.7 was employed by previous systematic reviews ^3–5^;
- For the primary outcome, we conducted a sensitivity analysis to allow for uncertainty caused by missing outcome data, as recommended for meta-analysis with missing data for binary variables.^6–8^ We assumed that the odds of an event occurring in the missing participants were half of those of the observed participants, meaning the Informative Missingness Odds Ratios (IMORs) were 0.5.^9^ Additionally, we allowed the IMOR to range from 0.5 to 1.5, as suggested as a plausible range for IMORs to define the uncertainty about the missing data.^9^

We could not explore the robustness of findings by excluding studies at high risk of bias because only one study was judged to have some concerns; the remaining studies were judged to be at high risk of bias.

*Sub-group analysis and investigation of heterogeneity*

There were too few studies for subgroup analyses of primary outcomes. Subgroup analyses by the duration of intervention were conducted for secondary outcomes; however, there was insufficient data for meta-analysis by type of HDP experienced during pregnancy, postpartum period at enrolment, by type of interventions (such as physical activity, diet), by single or combined interventions, by reproductive age (≤49 vs. >49 years old), and by settings (low- and middle-income countries vs. high-income countries).

*Post-hoc analysis*

For the study that followed up with their participants for a longer period after the initial intervention ended, we included data from the last follow-up in a meta-analysis. For outcomes that one or two studies with outlying results may cause bias, a post-hoc sensitivity analysis was conducted to explore the influence of studies on the overall pooled results by conducting a leave-one-out meta-analysis.^10,11^

**Appendix S5_Included studies and relevant studies**

**Reference list of included studies**

1. Cairns AE, Tucker KL, Leeson P, Mackillop LH, Santos M, Velardo C, Salvi D, Mort S, Mollison J, Tarassenko L, et al. Self-Management of Postnatal Hypertension: The SNAP-HT Trial. Hypertension (0194911X). 2018;72:425–432.
2. Hirshberg A, Downes K, Srinivas S. Comparing standard office-based follow-up with text-based remote monitoring in the management of postpartum hypertension: a randomised clinical trial. BMJ Quality & Safety. 2018;27:871–877.
3. Rich-Edwards JW, Stuart JJ, Skurnik G, Roche AT, Tsigas E, Fitzmaurice GM, Wilkins-Haug LE, Levkoff SE, Seely EW. Randomized Trial to Reduce Cardiovascular Risk in Women with Recent Preeclampsia. Journal of Women’s Health (15409996). 2019;28:1493–1504.
4. Hutchesson MJ, Taylor R, Shrewsbury VA, Vincze L, Campbell LE, Callister R, Park F, Schumacher TL, Collins CE. Be Healthe for Your Heart: A Pilot Randomized Controlled Trial Evaluating a Web-Based Behavioral Intervention to Improve the Cardiovascular Health of Women with a History of Preeclampsia. Int J Environ Res Public Health. 2020;17:5779.
5. Lewey J, Murphy S, Zhang D, Putt ME, Elovitz MA, Riis V, Patel MS, Levine LD. Effectiveness of a Text-Based Gamification Intervention to Improve Physical Activity Among Postpartum Individuals With Hypertensive Disorders of Pregnancy: A Randomized Clinical Trial. JAMA Cardiol. 2022;7:591–599.
6. Riemer M, Schulze S, Wagner L, Richter M, Ayerle G, Simm A, Seeger S, Schwesig R, Tchirikov M, Seliger G. Cardiovascular Risk Reduction in Women Following Hypertensive Disorders of Pregnancy – a Prospective, Randomised, Controlled Interventional Study. Geburtshilfe Frauenheilkd. 2021;81:966–978.
7. Parfenova M, Côté A-M, Cumyn A, Pesant M-H, Champagne M, Roy-Lacroix M-È, Malick M, Sauvé N. Impact of an Educational Pamphlet on Knowledge About Health Risks After Hypertensive Disorders of Pregnancy: A Randomized Trial. J Obstet Gynaecol Can. 2021;43:182–190.
8. Kitt JA, Fox RL, Cairns AE, Mollison J, Burchert HH, Kenworthy Y, McCourt A, Suriano K, Lewandowski AJ, Mackillop L, et al. Short-Term Postpartum Blood Pressure Self-Management and Long-Term Blood Pressure Control: A Randomized Controlled Trial. Hypertension (0194911X). 2021;78:469–479.
9. Kitt J, Fox R, Frost A, Shanyinde M, Tucker K, Bateman PA, et al. Long-Term Blood Pressure Control After Hypertensive Pregnancy Following Physician-Optimized Self-Management: The POP-HT Randomized Clinical Trial. JAMA. 2023 Nov 28;330(20):1991–9.
10. Nicklas JM, Pyle L, Soares A, Leiferman JA, Bull SS, Tong S, et al. The Fit After Baby randomized controlled trial: An mHealth postpartum lifestyle intervention for women with elevated cardiometabolic risk. PLOS ONE. 2024 Jan 9;19(1):e0296244.
11. Muijsers HEC, Wu P, van der Heijden OWH, Wijnberger LDE, van Bijsterveldt C, Buijs C, Pagels J, Tönnies P, Heiden S, Roeleveld N, et al. Home blood pressure monitoring detects unrevealed hypertension in women with a history of preeclampsia: Results of the BP-PRESELF study. Am J Prev Cardiol. 2022;12:100429.
12. Wang TL, Quinn BA, Hart R, Wiener AA, Facco FL, Simhan HN, Hauspurg AK. The effect of a neonatal sleep intervention on maternal postpartum hypertension: a randomized trial. Am J Obstet Gynecol MFM. 2024;6:101239.
13. Arkerson BJ, Finneran MM, Harris SR, Schnorr J, McElwee ER, Demosthenes L, Sawyer R. Remote Monitoring Compared With In-Office Surveillance of Blood Pressure in Patients With Pregnancy-Related Hypertension. Obstet Gynecol. 2023;142:855–861.
14. Hauspurg A, Seely EW, Rich-Edwards J, Hayduchok C, Bryan S, Roche AT, Jeyabalan A, Davis EM, Hart R, Shirriel J, et al. Postpartum home blood pressure monitoring and lifestyle intervention in overweight and obese individuals the first year after gestational hypertension or pre-eclampsia: A pilot feasibility trial. BJOG. 2023;130:715–726.
15. Ekawati E, Setyowati S, Budiati T. “Sehati” health education to improve physical and psychological adaptation of the postpartum women having pre-eclampsia. Enfermeria Clinica. 2019;29:199–204.
16. Hoppe KK, Thomas N, Zernick M, Zella JB, Havighurst T, Kim K, Williams M, Niu B, Lohr A, Johnson HM. Telehealth with remote blood pressure monitoring compared with standard care for postpartum hypertension. Am J Obstet Gynecol. 2020;223:585–588.

**Table S4. List of eligible studies involved participants with previous hypertensive disorder of pregnancy at baseline but did not report outcomes for these participants**

| **Author Year** | **Title** | **Journal and doi** |
| --- | --- | --- |
| Berks 2019 | Feasibility and effectiveness of a lifestyle intervention after complicated pregnancies to improve risk factors for future cardiometabolic disease | Pregnancy Hypertension https://doi.org/10.1016/j.preghy.2018.12.004 |
| Miremberg 2022 | Smartphone-based counseling and support platform and the effect on postpartum lactation: a randomized controlled trial | American Journal of Obstetrics & Gynecology MFM  https://doi.org/10.1016/j.ajogmf.2021.100543 |
| Clapp 2024 | Postpartum Primary Care Engagement Using Default Scheduling and Tailored Messaging | JAMA Network  doi:10.1001/jamanetworkopen.2024.22500 |

**Table S5. List of studies that finished their trials but have not published their findings**

| **Author Year** | **Title** | **Registration number** |
| --- | --- | --- |
| Henry 2020 | Blood pressure postpartum (BP2) RCT: Follow-up and lifestyle behaviour change strategies in the first 12 months after hypertensive pregnancy | ACTRN12618002004246 |
| Lewkowitz 2023 | App-based Remote Blood Pressure Monitoring | NCT05595629 |

**Table S6. List of excluded full texts**

| **Author Year** | **Title** | **Reasons for exclusion** |
| --- | --- | --- |
| Lohr 2023 | Does Daily Self-Weighing Contribute to Postpartum Weight Loss? A Secondary Analysis of Daily Postpartum Weights among Women with Hypertensive Disorders of Pregnancy. | Wrong study design |
| Ugurlu 2021 | The Effect of an Education and Counseling Program on Maternal/Neonatal Outcomes in Pregnant Women at Risk of Preeclampsia. | Wrong patient population |
| Ziesler 2021 | Low physical activity levels 1 year after pregnancy complications. | Wrong study design |
| Pealing 2019 | A randomised controlled trial of blood pressure self-monitoring in the management of hypertensive pregnancy. OPTIMUM-BP: A feasibility trial. | Wrong outcomes |
| Jiang 2019 | The efficacy of the Dietary Approaches to Stop Hypertension diet with respect to improving pregnancy outcomes in women with hypertensive disorders. | Wrong intervention |
| Sammel 2019 | Text message remote monitoring reduced racial disparities in postpartum blood pressure ascertainment. | Wrong comparator |
| Scholten 2015 | Aerobic Exercise Training in Formerly Preeclamptic Women: Effects on Venous Reserve. | Wrong intervention |
| Thornton 2009 | Perinatal outcomes in nutritionally monitored obese pregnant women: a randomized clinical trial. | Wrong patient population |
| Knuist 1998 | Low sodium diet and pregnancy-induced hypertension: a multi-centre randomised controlled trial. | Wrong patient population |
| Reyes 2012 | Early intensive obstetric and medical nutrition care is associated with decreased prepregnancy obesity impact on perinatal outcomes. | Wrong patient population |
| Soya 2003 | Self-care activities of pregnancy induced hypertension and maternal outcome. | Wrong patient population |
| Daley 2021 | Practice nurse-supported weight self-management delivered within the national child Immunisation programme for postnatal women: a feasibility cluster RCT | Wrong patient population |
| VonSternberg 2020 | Processes of change as mediators of an intervention to prevent alcoholand tobacco-exposed pregnancy | Wrong patient population |
| Harrison 2014 | How effective is self-weighing in the setting of a lifestyle intervention to reduce gestational weight gain and postpartum weight retention? | Wrong patient population |
| DietzdeLoos 2022 | Pregnancy Rate and Outcomes Following a Randomized Controlled Three-component Lifestyle Intervention in Women with PCOS | Wrong patient population |
| Daley 2020 | Feasibility and acceptability of a brief routine weight management intervention for postnatal women embedded within the national child immunisation programme in primary care: randomised controlled cluster feasibility trial | Wrong patient population |
| Altazan 2019 | Mood and quality of life changes in pregnancy and postpartum and the effect of a behavioral intervention targeting excess gestational weight gain in women with overweight and obesity: a parallel-arm randomized controlled pilot trial | Wrong patient population |
| Karamali 2014 | The effects of DASH diet on lipid profiles and biomarkers of oxidative stress in overweight and obese women with polycystic ovary syndrome: a randomised clinical trial | Wrong patient population |
| Harrison 2011 | Limiting excess weight gain in high-risk pregnancies: a randomized controlled trial | Wrong patient population |
| O'Reilly 2021 | Bump 2 Baby and Me: a study to test health coaching for healthy eating and activity during pregnancy and the first year after a baby is born | On-going study |
| Arentz 2017 | Randomized controlled trial of combined lifestyle and herbal medicine in women with polycystic ovary syndrome | Wrong patient population |
| Lewey 2022 | Remote Behavioral Weight Loss Intervention for Postpartum Mothers With Cardiovascular Risk Factors | Conference presentation |
| Gordon 2020 | Pre-babe: impact of pre-conception weight loss for women above a healthy weight | Wrong patient population |
| Parrish 2016 | Processes of change in preventing alcohol exposed pregnancy: a mediation analysis | Wrong patient population |
| Zhao 2019 | Effects of antenatal depression screening and intervention among Chinese high-risk pregnant women with medically defined complications: a randomized controlled trial | Wrong intervention |
| Fox 2021 | Short term postpartum blood pressure management and long-term blood pressure control: a randomised controlled trial | Conference presentation |
| Arkerson 2022 | Pregnancy-Related Hypertension: adherence to a New Type of Monitoring (PHANTOM) | Protocol |
| Henry 2019 | Early intervention to reduce cardiovascular risk after hypertensive pregnancy: the BP2 (Blood Pressure Postpartum) randomised trial | Conference presentation |
| Zhao 2017 | A randomized controlled trial: effects of a prenatal depression intervention on perinatal outcomes among Chinese high-risk pregnant women with medically defined complications | Wrong patient population |
| McManus 2017 | The snap-HT trial: self-management of antihypertensive medication postpartum - Can women do it better? | Conference presentation |
| Nagraj 2023 | A Mobile Clinical Decision Support System for High-Risk Pregnant Women in Rural India (SMARThealth Pregnancy): pilot Cluster Randomized Controlled Trial | Wrong intervention |
| Lara 2010 | Outcome results of a psycho-educational intervention in pregnancy to prevent PPD: a randomized control trial | Wrong patient population |
| Handlin 2012 | Influence of common birth interventions on maternal blood pressure patterns during breastfeeding 2 days after birth | Wrong patient population |
| Mejdoubi 2014 | Effects of nurse home visitation on cigarette smoking, pregnancy outcomes and breastfeeding: a randomized controlled trial | Wrong patient population |
| Kerver 2023 | Feasibility of a Food Delivery Intervention during Pregnancy in a Rural US Population: the PEAPOD Pilot Study | Wrong patient population |
| Brooten 2007 | Women with high-risk pregnancies, problems, and APN interventions: clinical scholarship | Wrong intervention |
| Zafman 2023 | A randomized trial of an interactive childbirth education platform in a high-risk population | Wrong patient population |
| Kim 2014 | Effects of Music Therapy and Phone Counseling on Postpartum Depression and Maternal Identity in High Risk Women | Wrong patient population |
| Cairns 2017 | A novel self-management intervention for adjustment of postnatal antihypertensive treatment | Conference presentation |
| Mclean 2022 | Lower Maternal Chronic Physiological Stress and Better Child Behavior at 18 Months: follow-Up of a Cluster Randomized Trial of Neonatal Intensive Care Unit Family Integrated Care | Wrong patient population |
| El-Mohandes 2011 | Smoking cessation and relapse among pregnant African-American smokers in Washington, DC | Wrong patient population |
| Spiegelman 2020 | 430: remote postpartum blood pressure surveillance for hypertensive disorders of pregnancy: a randomized clinical trial | Conference presentation |
| Triebwasser 2020 | 1077: effect of a text-based blood pressure monitoring program on continuity care after hypertensive pregnancy | Wrong study design |
| Shaw 2013 | Prevention of traumatic stress in mothers with preterm infants: a randomized controlled trial | Wrong patient population |
| Olsen 2022 | Effects of the healthy start randomized intervention on psychological stress and sleep habits among obesity-susceptible healthy weight children and their parents | Wrong patient population |
| Triebwasser 2022 | Nudge intervention to transition care after hypertensive disorders of pregnancy: a randomized clinical trial | Wrong intervention |
| Dodd 2022 | The effect of pre-pregnancy dietary advice and regular exercise to promote health in women with BMI between 18.5 and 24.9kg/m2, on pregnancy outcomes: the Begin Better 2.0 randomised trial | On-going study |
| Triebwasser 2020 | 221: successful implementation of remote blood pressure monitoring for postpartum hypertension | Wrong study design |
| Goulding 2023 | Breastfeeding initiation and duration among people with mild chronic hypertension: a secondary analysis of the Chronic Hypertension and Pregnancy (CHAP) trial | Wrong intervention |
| Hull 2020 | The effect of high dietary fiber intake on gestational weight gain, fat accrual, and postpartum weight retention: a randomized clinical trial | Wrong patient population |
| Jakicic 2017 | Effect of Wearable Technology Combined With a Lifestyle Intervention on Long-Term Weight Loss: the IDEA Randomized Clinical Trial | Wrong patient population |
| Barrera 2015 | Online prevention of postpartum depression for Spanish- and English-speaking pregnant women: a pilot randomized controlled trial | Wrong patient population |
| Oostingh 2019 | The coaching program 'Smarter Pregnancy' is the first effective m Health intervention to adopt healthy nutrition and lifestyle behaviours in subfertile couples: a randomised controlled trial | Wrong patient population |
| Okawa 2019 | Effect of continuum-of-care intervention package on improving contacts and quality of maternal and newborn healthcare in Ghana: a cluster randomised controlled trial | Wrong patient population |
| Dodd 2014 | Optimising gestational weight gain and improving maternal and infant health outcomes through antenatal dietary, lifestyle and exercise advice: the OPTIMISE randomised trial | Wrong patient population |
| Lane 2023 | Association of a Lifestyle Intervention with Blood Pressure Trajectories During Pregnancy and Postpartum in Women with Pre-pregnancy Overweight and Obesity | Wrong patient population |
| Hung 2014 | Self-monitoring and self-titration of antihypertensive medications result in better systolic blood pressure control | Wrong patient population |
| Rosborough 2017 | Effect of a high polyphenol intervention on Pregnancy Associated Plasma Protein-A in participants with hypertension | Wrong intervention |
| Vincent 2019 | Effect of breaking up sitting on health and cognition in sleep-restricted and non-sleep restricted individuals | Wrong intervention |
| Oken 2013 | Effects of an intervention to promote breastfeeding on maternal adiposity and blood pressure at 11.5 y postpartum: results from the Promotion of Breastfeeding Intervention Trial, a cluster-randomized controlled trial | Wrong patient population |
| Fard 2016 | Effect of lifestyle educational package on prevention of postpartum health problems in nulliparous mothers: a randomized clinical trial | Wrong patient population |
| Frank 1987 | Commercial discharge packs and breast-feeding counseling: effects on infant-feeding practices in a randomized trial | Wrong patient population |
| Haire-Joshu 2019 | Randomized Controlled Trial of Home-Based Lifestyle Therapy on Postpartum Weight in Underserved Women with Overweight or Obesity | Wrong patient population |
| Hoppu 2014 | Maternal dietary counseling reduces total and LDL cholesterol postpartum | Wrong patient population |
| O'Kane 2018 | Cow Milk Consumption Increases Iodine Status in Women of Childbearing Age in a Randomized Controlled Trial | Wrong patient population |
| Poskus 2021 | Preventing Hemorrhoids And Fissures Of Pregnancy: results Of The Randomized Controlled Trial | Wrong patient population |
| Tobon 2018 | Lasting Effects of Minding the BabyÂ® Home Visiting Program for Young Families | Conference presentation |
| Grote 2014 | Culturally relevant treatment services for perinatal depression in socio-economically disadvantaged women: the design of the MOMCare study | Wrong patient population |
| Rendall-Mkosi 2013 | A randomized controlled trial of motivational interviewing to prevent risk for an alcohol-exposed pregnancy in the Western Cape, South Africa | Wrong patient population |
| Crovetto 2022 | Effects of Mediterranean Diet or Mindfulness-Based Stress Reduction on Prevention of Small-for-Gestational Age Birth Weights in Newborns Born to At-Risk Pregnant Individuals: the IMPACT BCN Randomized Clinical Trial | Wrong patient population |
| McDuffie 1996 | Effect of frequency of prenatal care visits on perinatal outcome among low-risk women. A randomized controlled trial | Wrong patient population |
| Osman 2023 | Beetroot juice, exercise, and cardiovascular function in women planning to conceive. | Wrong patient population |
| Horn 2023 | Feasibility of a Postpartum Web- and Phone-Based Lifestyle Program for Women with a History of Preeclampsia or Gestational Diabetes: A Pilot Intervention Study. | Wrong comparator |
| Rasouli 2023 | Can integrating religiosity and spirituality into postpartum care improve the quality of life in women with preeclampsia. | Wrong intervention |
| Wen 2023 | Effects of information-knowledge-attitude-practice health education combined with cluster-based care in patients with gestational hypertension. | Wrong patient population |
| Ssetaala 2022 | Improving access to maternal health services among rural hard-to-reach fishing communities in Uganda, the role of community health workers. | Wrong patient population |
| vanKesteren 2015 | Prevention of cardiovascular risk in women who had hypertension during pregnancy after 36 weeks gestation. | No comparator |
| Leng 2023 | Antenatal mobile-delivered mindfulness-based intervention to reduce perinatal depression risk and improve obstetric and neonatal outcomes: A randomized controlled trial | Wrong patient population |
| Aderibigbe 2023 | Tight vs liberal control of mild postpartum hypertension: a randomized controlled trial | Wrong intervention |
| Wing 2023 | Preconception weight loss to improve pregnancy outcomes: Does the evidence justify national recommendations? | Wrong study design |
| Tangren 2018 | Can blood pressure self-monitoring improve postpartum management of pregnancy-associated hypertension? | Wrong study design |
| Peccei 2017 | Intensive Prenatal Nutrition Counseling in a Community Health Setting | Wrong patient population |
| Creighton 2014 | Are non-nutritive sweetened beverages comparable to water in weight loss trials? | No comparator |
| Kieffer 2013 | Effect of the Healthy MOMs Lifestyle Intervention on Reducing Depressive Symptoms Among Pregnant Latinas | Wrong patient population |
| Olayiwola 2013 | Living Smart, Living Fit: a patient-centered program to improve perinatal outcomes in a community health center population | Wrong patient population |
| Huang 2011 | A diet and physical activity intervention for preventing weight retention among Taiwanese childbearing women: A randomised controlled trial | Wrong patient population |
| Hayes 2004 | Prenatal depression: a randomized controlled trial in the emotional health of primiparous women | Wrong patient population |
| Lawrence 2003 | A cluster randomised controlled trial of smoking cessation in pregnant women comparing interventions based on the transtheoretical (stages of change) model to standard care | Wrong patient population |
| Tam 2003 | A randomised controlled trial of educational counselling on the management of women who have suffered suboptimal outcomes in pregnancy | Wrong patient population |
| Langer 1996 | The Latin American trial of psychosocial support during pregnancy: Effects on mother's wellbeing and satisfaction | Wrong patient population |

**Appendix S6_Additional information on characteristics of included studies**

**Table S7_Additional information of included studies**

| **Author Year** | **Intervention (I)** | **Control (C)** | **Entry n, (I/C)** | **Loss to follow-up n, (I/C)** | **Research Integrity** |
| --- | --- | --- | --- | --- | --- |
| Arkerson 2023^12^ | Participants were given a home BP monitor to self-monitor their BP and entered the readings into an app. The results were automatically uploaded to a secure web-based platform for remote review by designated physicians at each site. Participants in both the control and intervention groups with elevated BP (140/90 or higher) received a text-based survey to check for symptoms. An on-call operator contacted participants with severe symptoms or a second high BP reading (160/100 or higher). Participants with severe symptoms or high BP were advised to go to the obstetric triage unit. Otherwise, physicians reviewed their blood pressure daily. Participants with consistent high BP (150/100 or higher) were contacted by a physician to adjust their medication. | Participants were given the date and time of their postpartum appointment (within 10 days after discharge) at the time of discharge. | 96/101 | 0/0 | No concern |
| Cairns 2018^13^ | Participants self-monitored their BP once daily, at approximately the same time each day, using a validated automatic BP monitor, and entered it into their mobile phones, which automatically transmitted it to the study server. In response, the telemonitoring service provided automated replies that informed participants how to down titrate their medications, according to a prespecified individualised medication reduction schedule. If readings were high, according to an algorithm, participants were asked to contact their National Health Service care team immediately. Self-monitoring was continued while taking antihypertensive treatment and for 5 days after treatment was ceased. Once treatment was ceased, if the participants' BP remained within normal limits for 5 consecutive days, they were instructed to change to weekly self-monitoring until the trial was completed to ensure medication did not need to be restarted if readings moved out of the target range again. | Participants had their BP monitored by their community midwife and their antihypertensive medication adjusted by their general practitioner. | 45/46 | 5/4 | No concern |
| Ekawati 2019^14^ | The participants received health education on the second and fifth days of postpartum. The health education involved understanding pre-eclampsia, complications of pre-eclampsia, danger signs of pre-eclampsia, pre-eclampsia treatment at home, nutrition and lifestyle recommendations for patients with pre-eclampsia, relaxation techniques to reduce tension, how to breastfeed a baby properly, breastfeeding problems and ways to overcome them, and kangaroo methods. | Not specified | 30/30 | 0/0 | Not assessed |
| Hauspurg 2023^15^ | In the first intervention arm, participants received resources from the Heart Health 4 New Moms (HH4NM) website, a BP monitor, and instructions to measure their BP twice in the morning and twice in the evening daily for one week each month. They also received automated reminders via text messaging and summative information through the iHealth application, along with monthly summaries from study staff that outlined the number of measures provided, mean BP values, and comparisons with prior months. | Participants received access to the HH4NM website, which included publicly available information regarding cardiovascular risk associated with pre-eclampsia and gestational hypertension and lifestyle recommendations to prevent CVD from the American Heart Association and the Pre-eclampsia Foundation. | 100/48 | 19/0 | No concern |
| Hirshberg 2018^16^ | Participants were instructed to monitor their BP and send readings twice daily via text message. They received feedback based on a preprogrammed automated algorithm, and further care if needed, following the same outpatient algorithm used in the office. Instead of repeat office visits for severely elevated BP values, they were advised to continue sending BP readings through the platform. | Participants had their BP monitoring scheduled for office-based nursing visits 4-6 days after giving birth, and their care at this visit was determined based on a physician-developed algorithm. | 103/103 | 41/26 | No concern |
| Hoppe 2020^17^ | Participants were provided with a tablet and Bluetooth BP monitor to transmit vitals to a central database. Trained nurses then guided them to initiate or titrate antihypertensive treatment. | Participants were advised to follow standard care, including a clinic visit seven to ten days and six weeks postpartum. | 214/214 | 19/0 | Not assessed |
| Hutchesson 2020^18^ | Participants received online resources developed by the program related to healthy eating, physical activity, weight management, and stress management via the Be Healthe for Your Heart webpage. They also received weekly newsletter emails summarising the information given by the program resources related to a specific health behaviour topic. | Participants received an email with a link to the National Heart Foundation website and were provided with access to the Be Healthe for Your Heart intervention after completing the three-month follow-up appointment. | 16/15 | 3/4 | No concern |
| Kitt 2021^19^ | Participants self-monitored their BP once daily, at approximately the same time each day, using a validated automatic BP monitor, and entered it into their mobile phones, which automatically transmitted it to the study server. In response, the telemonitoring service provided automated replies that informed participants how to down titrate their medications, according to a prespecified individualised medication reduction schedule. If readings were high, according to an algorithm, participants were asked to contact their National Health Service care team immediately. Self-monitoring was continued while taking antihypertensive treatment and for 5 days after treatment was ceased. Once treatment was ceased, if the participants' BP remained within normal limits for 5 consecutive days, they were instructed to change to weekly self-monitoring until the trial was completed to ensure medication did not need to be restarted if readings moved out of the target range again. | Participants had their BP monitored by their community midwife and their antihypertensive medication adjusted by their general practitioner. Monitoring and medication managed by HC | 30/31^b^ | -/-^c^ | No concern |
| Kitt 2023^20^ |  | Participants were managed as per usual NHS led care with assessment by their own health care professionals and adjustment of their medications as is needed. The BP of this group will be monitored and recorded at the same time-points and in the same manner as the intervention arm as will all other secondary outcome measures. | 112/108 | 7/13 | No concern |
| Lewey 2021^21^ | Participants used a wearable device to track their daily step counts, received automated daily feedback on their progress towards their step goal, and participated in a team-based model of social incentives that gamified physical activity. | Participants used the same wearable device as the intervention group to track their daily step counts and received automated daily feedback on their progress towards their step goal. | 63/64 | 1/0 | No concern |
| Muijsers 2022^22^ | Participants were given a home BP monitor to self-monitor their BP for 7 consecutive days each month over one year. BP measurements were preferably taken twice a day, in the morning and evening. The results were automatically uploaded to an online patient health file. They received monthly feedback on their blood pressure measurements and were given lifestyle advice if necessary. In case of elevated blood pressure, participants were referred to their general practitioners. | Participants were not scheduled for BP measurements during follow-up but were requested to record any self-measured or doctor-measured BP readings. | 99/99 | 3/4 | No concern |
| Nicklas 2024^23^ | Participants received 12 weeks of daily evidence-based content, facilitated tracking of weight, diet, and activity, and included weekly coaching and gamification with points and rewards. | Participants gained access to the Text4baby app, which sends 2–4 free text messages weekly. These messages offer information on baby care and resources for women, customized to their number of weeks postpartum. | 54/28 | 15/9 | No concern |
| Parfenova 2021^24^ | After usual medical counselling, participants were given an educational pamphlet on hypertensive disorders of pregnancy, including causes, risk factors, risks for future pregnancies, long-term health implications such as cardiovascular diseases, and strategies to mitigate these risks. | The participants received the same educational pamphlet by email after the study was finished. | 57/56 | 11/4 | No concern |
| Rich-Edwards 2019^25^ | Participants received web-based educational and motivational modules (Be Healthe for your Heart) and personalised lifestyle coaching from a registered dietitian trained in patient-centred counselling to help them set and meet positive lifestyle goals. The modules included healthy eating, using the balanced plate and modelled on the DASH diet, increasing physical activity, and identifying promoters and barriers to adopting a healthy lifestyle. | Participants also received access to the web-based educational and motivational modules. | 76/75 | 7/5^a^ | No concern |
| Riemer 2021^26^ | Participants received 3 individual nutritional counselling sessions and a weekly cardiovascular exercise in 6 months to improve their aerobic endurance and overall strength under laboratory conditions. Additionally, participants were advised to take brisk walks every 2 to 3 days. | Participants received a single session of individual nutritional counseling. | 19/19 | 5/4 | No concern |
| Wang 2024^27^ | Participants were given the SNOO responsive bassinet and instructions on how to set it up and use the SNOO Smart Sleeper mobile app. They were asked to have their infant sleep in the SNOO-responsive bassinet as often as possible. | Participants received the current standard of care of safe sleep education in the postpartum period. | 54/56 | 9/25^a^ | No concern |
| ^a^Sample size for the last follow-up  ^b^The control group had 3 women who originally were not randomised in the SNAP-HT  ^c^101 consented for re-contact; however, the article did not specify the number in each group. | | | | | |

**Appendix S7_Risk of bias assessments**


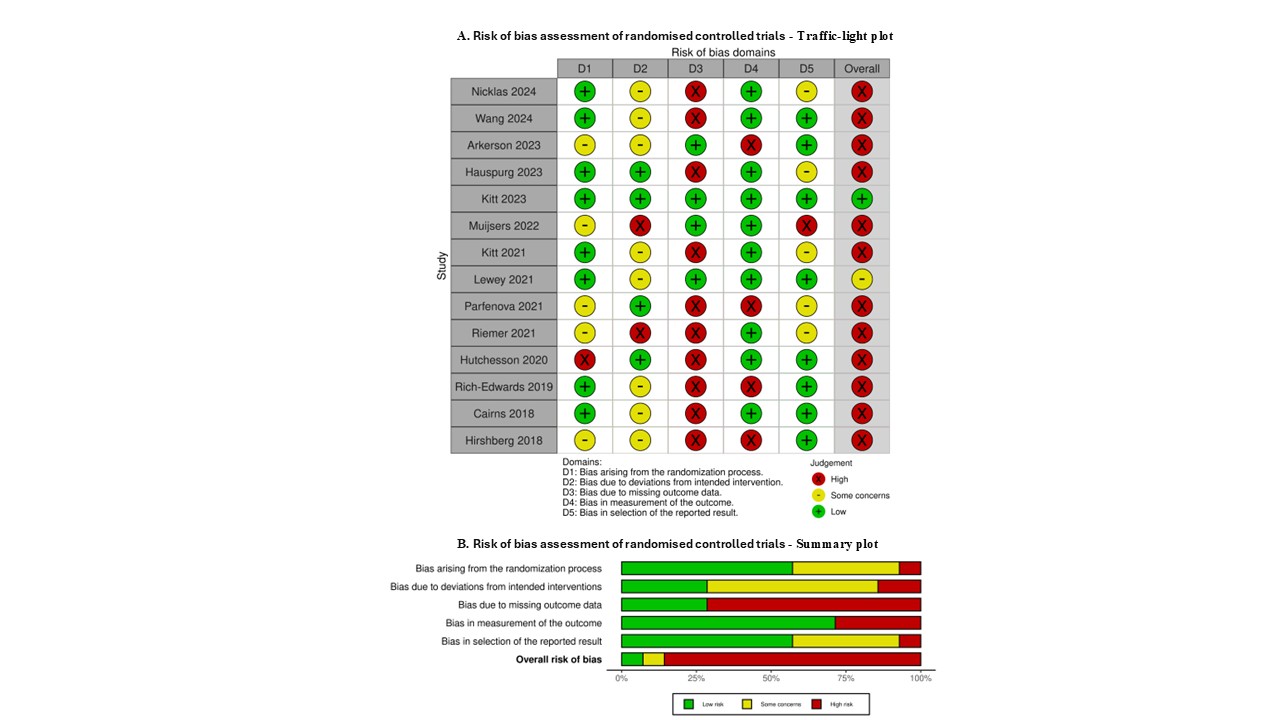


**Figure S1. Risk of bias assessment of randomised controlled trials included in this review (A) Traffic-light plot Summary plot and (B) Summary plot.**

**
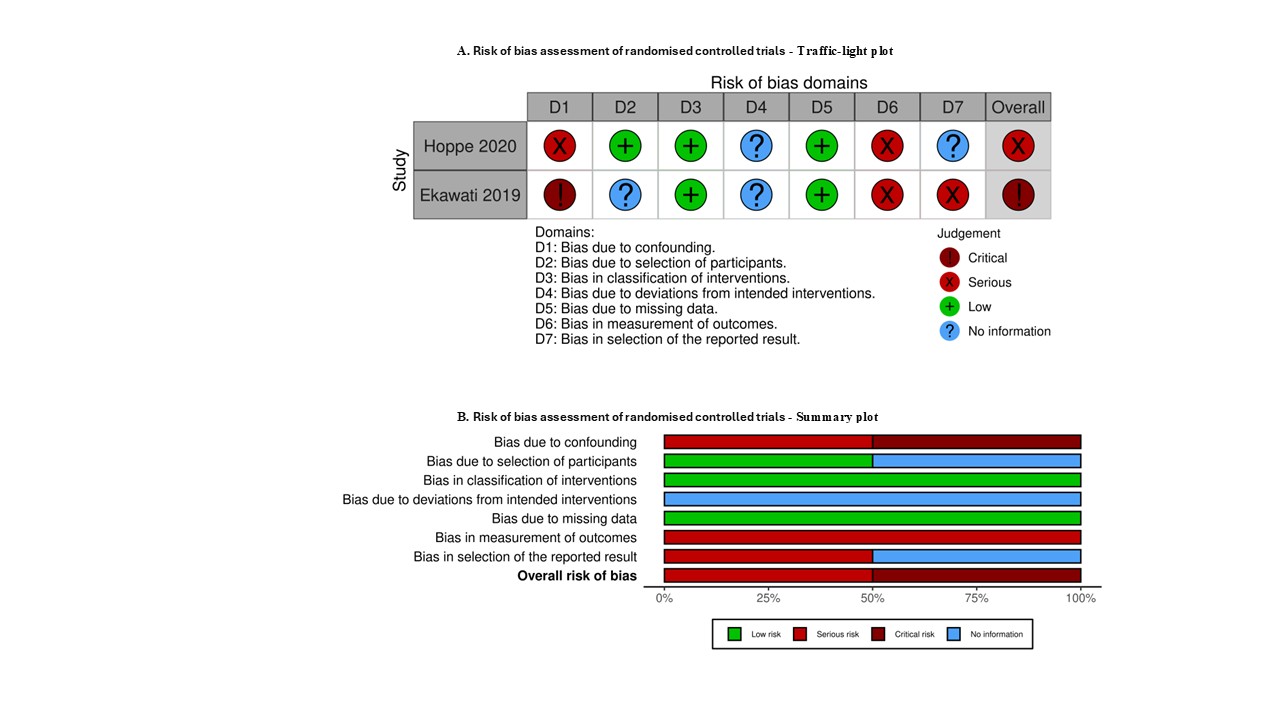
**

**Figure S2. Risk of bias assessment of non-randomised trials included in this review (A) Traffic-light plot Summary plot and (B) Summary plot.**

Twelve reports from eleven RCTs had a high risk of bias primarily due to missing outcome data,^18,27,26,24,15,13,16,25,19,23^ and bias in the measurement of the outcome.^12,16,24,25^ One RCT had some concerns,^21^ and one RCT had low risk of bias (Figure S7).^20^ One non-RCT had a critical risk of bias primarily due to the risk of confounding,^14^ and the other had a serious risk of bias due to confounding and outcome measurements (Figure 8).^17^

**Appendix S8_Secondary outcomes, sub-group analyses, sensitivity analyses and post-hoc analyses**

1. **Secondary outcomes**
   1. Use of anti-hypertensive medication

The effect of self-care interventions on the risk of using antihypertensive medications is uncertain (RR 1.20; 95% CI 0.82, 1.75; 7 trials; 1,063 women; *very low-certainty evidence*) (Figure S3, Table 2).


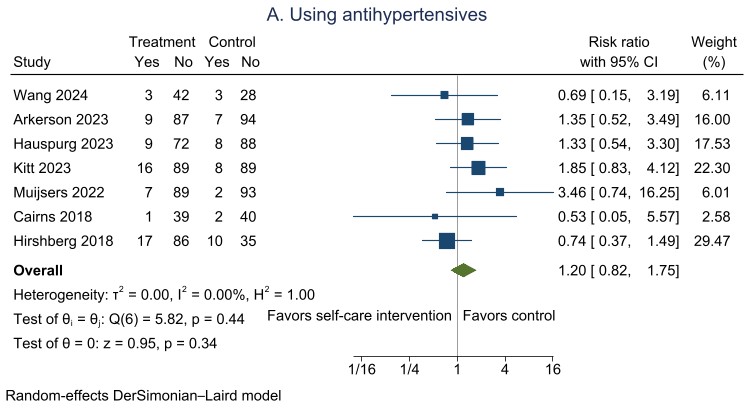


**Figure S3. The effects of self-care interventions on the risk of using antihypertensives**

- 1. Postpartum hypertension readmission, additional emergency hospital visits, body mass index, physical activity, total energy intake, and smoking

Meta-analysis of 3 trials involving 605 women showed that self-care interventions likely decrease the risk of postpartum hypertension readmission (RR 0.35; 95% CI 0.14, 0.89; *moderate-certainty evidence*) (Figure S4A, Table 2).

Pooled results showed that self-care interventions may make no difference to the risk of additional emergency department or office visits for hypertension not resulting in readmission (RR 2.22; 95% CI 0.93, 5.33; *low-certainty evidence*) (Figure S4B, Table 2).

We are uncertain about whether self-care interventions were associated with a decrease in body mass index (MD -0.46; 95% CI -1.17, 0.25 kg/m^2^; 24 trials; 503 women; *very* *low-certainty evidence*), an increase in the metabolic equivalent task (MET) (MD -528.67; 95% CI -502.53, 1559.87 MET mins/week; 2 trials; 170 women; *very low-certainty evidence*), a decrease in total energy intake (MD -241.07; 95% CI -720.59, 238.45 Kcal/day; 2 trials; 112 women; *very* *low-certainty evidence*), and a reduction in the risk of smoking (RR 1.39; 95% CI 0.5, 3.55; 2 trials; 393 women; *very* *low-certainty evidence*) (Figure S4C-F, Table 2).

One RCT (148 women) reported no association between self-care interventions and a reduction in BMI (p=0.70), with a median change of -0.4, 0.3, and -0.1 kg/m^2^ in intervention arm 1 (home BP monitoring and access to health education from an online website), intervention arm 2 (received all the interventions outlined in arm 1 and coaching sessions), and control arm.^15^


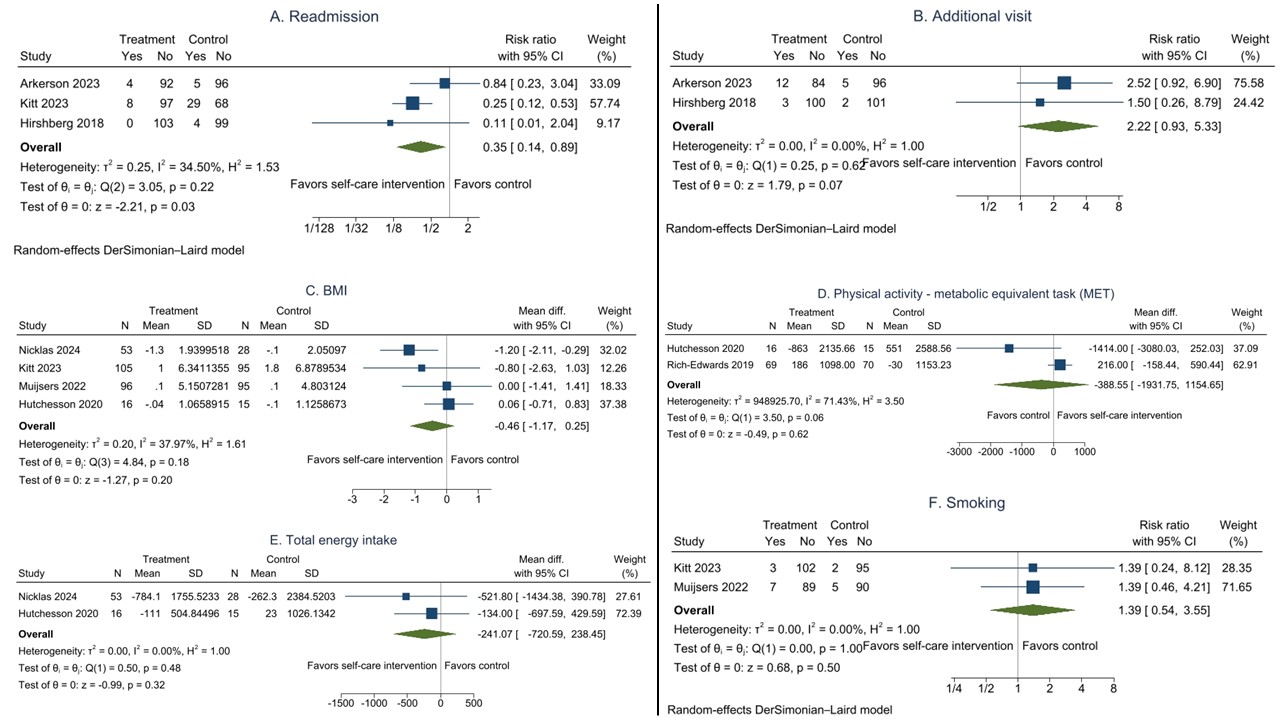


**Figure S4. The effects of self-care interventions on the risk of postpartum hypertension readmission (A), additional emergency hospital visits (B), on the mean difference from baseline body max index (C), metabolic equivalent task (D), total energy intake (E), and on the risk of smoking (F)**

One RCT (148 women) reported no association between self-care interventions and a reduction in BMI (p=0.70), with a median change of -0.4, 0.3, and -0.1 kg/m^2^ in intervention arm 1 (home BP monitoring and access to health education from an online website), intervention arm 2 (received all the interventions outlined in arm 1 and coaching sessions), and control arm.^15^

One trial involving 148 women reported no association between self-care interventions and an increase in metabolic equivalent task (MET) s/week (p=0.74), with a median change of -3.5, -9.4, -13.0 METs/week in intervention arm 1 (home BP monitoring and access to health education from an online website), intervention arm 2 (received all the interventions outlined in arm 1 and coaching sessions), and control arm.^15^

- 1. Daily step count

One trial involving 127 women, reported that the MD of daily step counts was 647 steps higher in the self-care group than in the control group, (adjusted mean difference (MD) 647; 95% CI 169, 1124 steps; p=0.01).^21^

- 1. Self-efficacy

One trial involving 139 women found that self-care interventions increased the self-efficacy score of eating habits (p=0.005), with a mean change of -0.1 in the self-care group and 0.1 units in the control group.^25^ However, the study reported no association between self-care interventions and an increase in self-efficacy score of physical activity (p=0.20), with a mean change of -0.1 in the self-care group and 0.2 units in the control group.^25^

One trial involving 148 women reported that self-care interventions increased the self-efficacy score of eating habits (p=0.04), with a median change of -0.17, 0.08, -0.17 units in intervention arm 1 (home BP monitoring and access to health education from an online website), intervention arm 2 (received all the interventions outlined in arm 1 and coaching sessions), and control arm.^15^ The study also reported no association between self-care interventions and an increase in self-efficacy score of physical activity (p=0.36), with a median change of 0.15, 0.00, -0.10 units in intervention arm 1 (home BP monitoring and access to health education from an online website), intervention arm 2 (received all the interventions outlined in arm 1 and coaching sessions), and control arm.^15^

- 1. Diabetes

One trial involving 191 women reported no association between self-care interventions and a reduction in the risk of diabetes, with 2.1% (2/96 women) in the self-care group and 1.1% (1/95 women) in the control group, (RR 1.96; 95% CI 0.18, 21.25; p=0.57).^22^

- 1. Total cholesterol

One trial involving 31 women found no association between self-care interventions and a reduction in total cholesterol (MD -0.5; 95% CI -0.2, 1.2 mmol/L), with a mean change of 0.01 mmol/L in the self-care group and 0.5 mmol/L in the control group.^18^

- 1. Sodium intake

One trial involving 31 women found no association between self-care interventions and a reduction in sodium intake (MD -361; 95% CI -32, 754), with a mean change of -94 mg in the self-care group and 267 mg in the control group.^18^

- 1. Quality of Life

One trial (31 women) using the Quality of Life Enjoyment and Satisfaction Questionnaire Short Form, found no association between self-care interventions and an increase in quality of life enjoyment and satisfaction score (MD 1.2; 95% CI -12.3, 9.9), with a mean change of 5.7% in the self-care group and 4.5% in the control group.^18^

Another trial (82 women) using the EQ-5D-5L VAS scale, found no association between self-care interventions and an increase in quality of life, (MD -3.3; 95% CI -7.5, 0.9).^13^ However, a trial involving 156 women EQ-5D-5L indexed values, found that self-care interventions increased the quality of life (MD 0.04; 95% CI 0.001, 0.08), with a mean change of 0.92 in the self-care group and 0.88 units in the control group.^20^

- 1. Depression, Anxiety, and Stress Scale (DASS scale)

One trial (31 women) using the DASS scale found no association between self-care interventions and a reduction in depression (MD 1.5; 95% CI -4.5, 1.6), anxiety (MD 1.9; 95% CI -5.9, 2.1), and stress (MD 1.2; 95% CI -5.2, 2.7).^18^

- 1. Breastfeeding

One trial involving 202 women found no association between self-care interventions and an increase in breastfeeding with 54.8% (57/105 women) in the self-care group and 43.9% (43/97 women) in the control group, (RR 1.22; 95% CI 0.70, 2.13).^20^

1. **Studies that did not contribute to review outcomes**

One RCT involving 113 women from Canada, using unvalidated Likert scales, reported global anxiety, anxiety about subsequent pregnancies, and anxiety about future CVD risks.^24^ One RCT involving 60 women from Indonesia reported physiological and psychological adaptation abilities.^14^

1. **Sensitivity analysis**
   1. Sensitivity analysis using a different correlation (r = 0.7) to impute missing SD for changes from baseline

The sensitivity analyses were conducted for two continuous outcomes involving studies with missing SDs for changes from baseline.

**Table S8. Sensitivity analyses replacing correlation coefficient values from r=0.5 in primary analyses to r=0.7 in studies with missing SD for changes from baseline**

|  | Correlation coefficient r=0.5 | | | Correlation coefficient r = 0.7 | | |
| --- | --- | --- | --- | --- | --- | --- |
|  | MD | 95% CI | I^2^(%) | MD | 95% CI | I^2^(%) |
| SBP (mmHg) | -3.24 | -5.42, -1.06 | 40.67 | -3.41 | -5.49, -1.33 | 53.42 |
| DBP (mmHg) | -3.07 | -4.88 -1.25 | 50.91 | -3.17 | -4.95, -1.40 | 63.88 |
| SBP: systolic blood pressure; DBP: diastolic blood pressure; MD: mean change from baseline | | | | | | |

From the sensitivity analysis, women receiving self-care interventions had lower systolic BP than women in the control group (9 trials; 952 women; MD from baseline -3.41, 95% CI -5.49, -1.33) (Table S8.1).

From the sensitivity analysis, women receiving self-care interventions had lower diastolic BP than women in the control group (9 trials; 952 women; MD from baseline -3.17, 95% CI -4.95, -1.40) (Table S8.1).

1. **Post-hoc analysis**
   1. Post-hoc analyses including data from the last follow-up of a study that followed up with their participants for a longer period after the initial intervention ended

One trial followed up with their trial participants at 3 years postpartum and reported systolic and diastolic BP.^19^ We included the data reported in year 3 to account for long-term follow-up. Data at 26 weeks of follow-up^13^ was replaced with data at year 3 follow-up^19^ to avoid double counting of participants.

**Table S9. Post-hoc analyses with data from the last follow-up**

|  | Primary analysis^*^ | | | Post-hoc analysis^†^ | | |
| --- | --- | --- | --- | --- | --- | --- |
|  | MD | 95% CI | I^2^ | MD | 95% CI | I^2^ (%) |
| SBP (mmHg) | -3.24 | -5.42, -1.06 | 40.67 | -3.52 | -5.93, -1.10 | 50.60 |
| DBP (mmHg) | -3.07 | -4.88 -1.25 | 50.91 | -3.27 | -5.30, -1.24 | 60.32 |
| SBP: systolic blood pressure; DBP: diastolic blood pressure; MD: mean change from baseline  ^*^9 trials involving 952 women, data at 26 weeks of follow-up of the SNAP-HT trial (Cairns 2018)  ^†^9 trials involving 931 women, data at year 3 of follow-up of the SNAP-HT trial (Kitt 2021) | | | | | | |

Pooled results in the post-hoc analysis from 9 trials involving 931 women showed that, compared to the control group, women receiving self-care interventions had lower systolic BP (MD from baseline --3.52, 95% CI 5.93, -1.10) and lower diastolic BP (MD from baseline -3.27, 95% CI -5.30, -1.24) (Table S8.2).

- 1. Post-hoc sensitivity analyses using leave-one-out meta-analysis

Compared to other studies in the meta-analyses of blood pressure, the Riemer 2021, Kitt 2023, and Muijsers 2022 study appear to have driven the pooled estimate to a significant extent due to their effect size, wide CIs, and sample size. Additionally, Nicklas 2024 and Hutchesson 2020 have a positive mean difference, which is the opposite effect direction to other studies. Therefore, we conducted leave-one-out analyses to assess each study's influence on the pooled results and heterogeneity.

All the overall effect sizes from the leave-one-out meta-analysis are close to the overall effect-size vertical line, and the magnitude of the mean difference remains negative (results favouring self-care interventions), their CI lines intersect with the vertical red line based on all the studies and p-values ≤ 0.01. This indicates that no studies substantially influence the overall results of our meta-analyses.


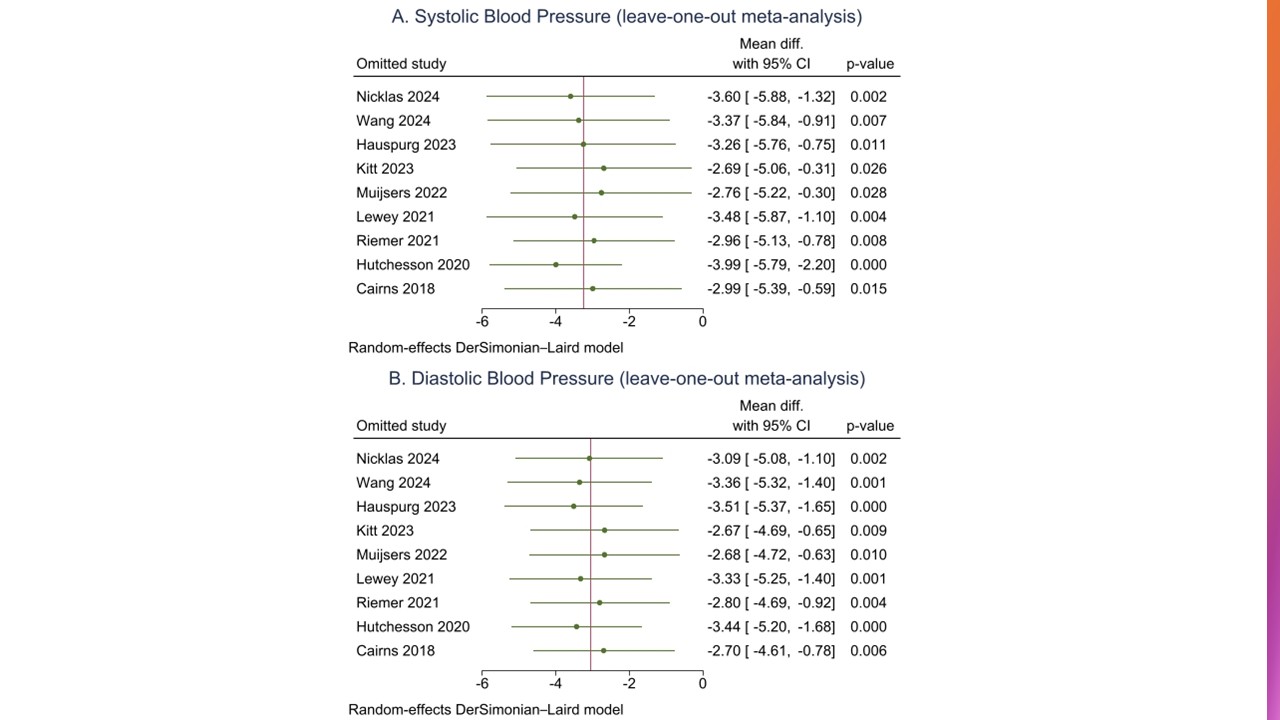


**Figure S5. The leave-one-out meta-analysis forest plots for the effects of self-care interventions on systolic blood pressure (A) and diastolic blood pressure (B)**

**References for Supplementary Materials**

1. Higgins J, Thomas J, Chandler J, Cumpston M, Li T, Page M, et al. Cochrane Handbook for Systematic Reviews of Interventions version 6.4 (updated August 2023) [Internet]. 2023 [cited 2023 Aug 22]. Available from: https://training.cochrane.org/handbook/current

2. Follmann D, Elliott P, Suh I, Cutler J. Variance imputation for overviews of clinical trials with continuous response. J Clin Epidemiol. 1992 Jul;45(7):769–73.

3. Berlim MT, McGirr A, Rodrigues dos Santos N, Tremblay S, Martins R. Efficacy of theta burst stimulation (TBS) for major depression: An exploratory meta-analysis of randomized and sham-controlled trials. Journal of Psychiatric Research. 2017 Jul 1;90:102–9.

4. Papadopoulos VP, Apergis N, Filippou DK. Nocturia in CPAP-Treated Obstructive Sleep Apnea Patients: a Systematic Review and Meta-Analysis. SN Compr Clin Med. 2020 Dec 1;2(12):2799–807.

5. Yagiz G, Akaras E, Kubis HP, Owen JA. The Effects of Resistance Training on Architecture and Volume of the Upper Extremity Muscles: A Systematic Review of Randomised Controlled Trials and Meta-Analyses. Applied Sciences. 2022 Jan;12(3):1593.

6. Mavridis D, White IR. Dealing with missing outcome data in meta-analysis. Research Synthesis Methods. 2020;11(1):2–13.

7. White IR, Higgins J. Meta-Analysis with missing data. Meta-Analysis in Stata: An Updated Collection from the Stata Journal, Second Edition [Internet]. STATA. 2016 [cited 2024 Jun 7]. Available from: https://www.stata.com/bookstore/meta-analysis-in-stata/

8. Mavridis D, Chaimani A. Allowing for uncertainty due to missing outcome data in meta-analysis [Internet]. Cochrane. 2018 [cited 2024 Jun 7]. Available from: https://training.cochrane.org/resource/allowing-uncertainty-due-missing-outcome-data-meta-analysis

9. Kahale LA, Khamis AM, Diab B, Chang Y, Lopes LC, Agarwal A, et al. Potential impact of missing outcome data on treatment effects in systematic reviews: imputation study. BMJ. 2020 Aug 26;370:m2898.

10. Meng Z, Wang J, Lin L, Wu C. Sensitivity analysis with iterative outlier detection for systematic reviews and meta-analyses. Statistics in Medicine. 2024;43(8):1549–63.

11. Cumpston M, Li T, Page MJ, Chandler J, Welch VA, Higgins JP, et al. Updated guidance for trusted systematic reviews: a new edition of the Cochrane Handbook for Systematic Reviews of Interventions. Cochrane Database Syst Rev. 2019 Oct 3;2019(10):ED000142.

12. Arkerson BJ, Finneran MM, Harris SR, Schnorr J, McElwee ER, Demosthenes L, et al. Remote Monitoring Compared With In-Office Surveillance of Blood Pressure in Patients With Pregnancy-Related Hypertension. Obstet Gynecol. 2023 Oct;142(4):855–61.

13. Cairns AE, Tucker KL, Leeson P, Mackillop LH, Santos M, Velardo C, et al. Self-Management of Postnatal Hypertension: The SNAP-HT Trial. Hypertension (0194911X). 2018 Aug;72(2):425–32.

14. Ekawati E, Setyowati S, Budiati T. “Sehati” health education to improve physical and psychological adaptation of the postpartum women having pre-eclampsia. Enfermeria Clinica. 2019 Jan 1;29:199–204.

15. Hauspurg A, Seely EW, Rich-Edwards J, Hayduchok C, Bryan S, Roche AT, et al. Postpartum home blood pressure monitoring and lifestyle intervention in overweight and obese individuals the first year after gestational hypertension or pre-eclampsia: A pilot feasibility trial. BJOG. 2023 Jun;130(7):715–26.

16. Hirshberg A, Downes K, Srinivas S. Comparing standard office-based follow-up with text-based remote monitoring in the management of postpartum hypertension: a randomised clinical trial. BMJ Quality & Safety. 2018 Nov;27(11):871–7.

17. Hoppe KK, Thomas N, Zernick M, Zella JB, Havighurst T, Kim K, et al. Telehealth with remote blood pressure monitoring compared with standard care for postpartum hypertension. Am J Obstet Gynecol. 2020 Oct;223(4):585–8.

18. Hutchesson MJ, Taylor R, Shrewsbury VA, Vincze L, Campbell LE, Callister R, et al. Be Healthe for Your Heart: A Pilot Randomized Controlled Trial Evaluating a Web-Based Behavioral Intervention to Improve the Cardiovascular Health of Women with a History of Preeclampsia. Int J Environ Res Public Health. 2020 Aug 10;17(16):5779.

19. Kitt JA, Fox RL, Cairns AE, Mollison J, Burchert HH, Kenworthy Y, et al. Short-Term Postpartum Blood Pressure Self-Management and Long-Term Blood Pressure Control: A Randomized Controlled Trial. Hypertension (0194911X). 2021 Aug;78(2):469–79.

20. Kitt J, Fox R, Frost A, Shanyinde M, Tucker K, Bateman PA, et al. Long-Term Blood Pressure Control After Hypertensive Pregnancy Following Physician-Optimized Self-Management: The POP-HT Randomized Clinical Trial. JAMA. 2023 Nov 28;330(20):1991–9.

21. Lewey J, Murphy S, Zhang D, Putt ME, Elovitz MA, Riis V, et al. Effectiveness of a Text-Based Gamification Intervention to Improve Physical Activity Among Postpartum Individuals With Hypertensive Disorders of Pregnancy: A Randomized Clinical Trial. JAMA Cardiol. 2022 Jun 1;7(6):591–9.

22. Muijsers HEC, Wu P, van der Heijden OWH, Wijnberger LDE, van Bijsterveldt C, Buijs C, et al. Home blood pressure monitoring detects unrevealed hypertension in women with a history of preeclampsia: Results of the BP-PRESELF study. Am J Prev Cardiol. 2022 Nov 11;12:100429.

23. Nicklas JM, Pyle L, Soares A, Leiferman JA, Bull SS, Tong S, et al. The Fit After Baby randomized controlled trial: An mHealth postpartum lifestyle intervention for women with elevated cardiometabolic risk. PLOS ONE. 2024 Jan 9;19(1):e0296244.

24. Parfenova M, Côté AM, Cumyn A, Pesant MH, Champagne M, Roy-Lacroix MÈ, et al. Impact of an Educational Pamphlet on Knowledge About Health Risks After Hypertensive Disorders of Pregnancy: A Randomized Trial. J Obstet Gynaecol Can. 2021 Feb;43(2):182–90.

25. Rich-Edwards JW, Stuart JJ, Skurnik G, Roche AT, Tsigas E, Fitzmaurice GM, et al. Randomized Trial to Reduce Cardiovascular Risk in Women with Recent Preeclampsia. Journal of Women’s Health (15409996). 2019 Nov;28(11):1493–504.

26. Riemer M, Schulze S, Wagner L, Richter M, Ayerle G, Simm A, et al. Cardiovascular Risk Reduction in Women Following Hypertensive Disorders of Pregnancy – a Prospective, Randomised, Controlled Interventional Study. Geburtshilfe Frauenheilkd. 2021 Aug;81(8):966–78.

27. Wang TL, Quinn BA, Hart R, Wiener AA, Facco FL, Simhan HN, et al. The effect of a neonatal sleep intervention on maternal postpartum hypertension: a randomized trial. Am J Obstet Gynecol MFM. 2024 Feb;6(2):101239.

**Appendix S9_Protocol**

1. **Background**
   1. Description of the condition

*Hypertensive disorders of pregnancy*

Hypertensive disorders of pregnancy (HDP) refer to a group of high blood pressure conditions, including gestational hypertension, preeclampsia/eclampsia, pre-existing (chronic) hypertension, and preeclampsia superimposed on chronic hypertension ^29^. The diagnostic criteria for hypertension in pregnancy are systolic blood pressure ≥140 mm Hg and/or diastolic blood pressure ≥90 mm Hg ^30^ at two separate measurements ^31^. The risk factors contributing to the development of HDP include a family history of HDP, pre-existing medical conditions (such as diabetes mellitus, pre-existing hypertension, autoimmune disease, and antiphospholipid syndrome), smoking, multiple pregnancies, and high body mass index ^32^.

*Global burden of hypertensive disorders of pregnancy*

Globally, HDP affects 18.1 million women yearly ^33^ and has a prevalence of 116.4 per 100,000 reproductive-aged women, varying by region ^34^. HDP is most prevalent in Africa, with a rate of 335 per 100,000 reproductive-aged women, followed by Southeast Asia and the Middle East, with a rate of 136.8 and 121.4 per 100,000 reproductive-aged women, respectively ^34^. The Western Pacific region has the lowest rate of 16 per 100,000 reproductive-aged women ^34^. Due to the wide range of health consequences that HDP can lead to, as well as its high prevalence, it is one of the most common causes of maternal morbidity and mortality ^35,36^, resulting in approximately 27,800 (95% UI 24,300 to 27,800) deaths worldwide in reproductive-aged women ^33^. The majority of these maternal deaths attributable to HDP occur in low- and middle-income countries, where health service utilisation is often lower than hoped, and high-quality maternal care is not routinely accessible for many women. For example, in 2019, it was estimated that the majority of HDP deaths occurred in countries with low socio-demographic index of 13,500 (95% UI 11,400 to 15,800) and low-middle socio-demographic index of 9,900 (95% UI 8,400 to 11,400) ^33^. HDP affects roughly 5.2% to 8.2% of all pregnant women, with the most common being pre-eclampsia ^37^, leading to increased risk of death, stroke, a caesarean delivery, renal insufficiency, liver dysfunction, acute kidney injury, or multimorbidity ^32,38^. HDP can also negatively affect fetuses and infants, leading to small-for-gestational-age infants, stillbirth, low birth weight, preterm birth seizures, and neonatal mortality ^38^.

*Hypertensive disorders of pregnancy and cardiovascular diseases*

In addition to the short-term pregnancy-related consequences of HDP, increasing evidence suggests an association between HPD and adult cardiovascular disease (CVD) in later life ^39^. Studies have indicated a 2 to 4-fold increase in risk of CVD in women who experienced HDP, such as increased risk of hypertension (RR 3.46, 95% CI: 2.67-4.49), heart failure (RR 2.53, 95% CI: 1.28-5.00, and ischemic heart disease (RR 2.06, 95% CI: 1.38-3.08) ^40^ that persists for at least a decade ^39^. CVDs encompass a range of heart and blood vessel disorders, from coronary heart disease and cerebrovascular disease to peripheral arterial disease, rheumatic heart disease, congenital heart disease, deep vein thrombosis and pulmonary embolism ^41^. These diseases are among the leading causes of death globally, accounting for 17.9 million deaths annually, at least 75% of which occurred in low- and middle-income countries ^41^.

Several hypotheses have been proposed on how HDP can lead to CVD in later life. One hypothesis is that HDP and subsequent CVD share common predisposing risk factors (such as type 2 diabetes, hypercholesterolemia, and increased body mass index) and manifest the same pathophysiologic processes at different times in a woman's life ^42,43^. Another suggested mechanism is due to cardiac and coronary changes. During pregnancy, the body undergoes cardiac and coronary changes to ensure sufficient oxygen supply to the placenta. However, sometimes, the body fails to fully recover from these changes (such as an increased left ventricular wall thickness or impaired left ventricular relaxation), leading to long-term damage that may result in CVD later in life ^44^. Additionally, poor cardiovascular adaptation during pregnancy can cause placental ischemia, leading to increased placental debris in the maternal circulation and endothelial dysfunction that persists after delivery, potentially leading to CVD risks later in life ^44^.

While CVD causes a higher mortality rate among women than men ^45^, it remains under-recognised, under-diagnosed, understudied, and under-treated in women ^46^. CVD is a major contributor to mortality in women, causing 35% of female deaths each year ^46^. Therefore, women with HDP may benefit from interventions aimed at screening for CVD risk factors and reducing such risks ^47^.

- 1. Description of self-care and self-care interventions

Self-care as defined by the World Health Organization, refers to an individual’s ability to maintain health, prevent disease, and cope with disability and illness with or without the assistance of health professionals ^48^. Self-care theoretically involves self-care maintenance, monitoring, and management ^49^. Self-care maintenance entails adopting behaviours that promote physical and emotional well-being, whether these behaviours are self-chosen or recommended by healthcare professionals ^49^. Self-care monitoring involves observing oneself for any changes in signs and symptoms, while self-care management refers to an appropriate response to any such changes ^49^. For instance, research has shown that women with HDP who self-monitor their blood pressure at home have 10% fewer hypertension-relation hospital admissions than those who do not (2.9% versus 13.5%, p = 0.004) ^50^.

Self-care interventions refer to tools to promote or support individuals’ ability to take care of themselves ^48^. For chronic diseases, it is suggested that self-care interventions should prioritise behaviour modification by equipping people with the knowledge and skills needed to actively participate in and manage their self-care maintenance, monitoring, and illness management ^51^. Self-care can be effective in diverse settings, but it becomes even more essential in resource-constrained settings where individuals may not have access to healthcare due to unaffordability or unavailability ^48^. This is especially important for groups that experience health inequities, such as ethnic minorities, people with low socioeconomic status, and women who lack social, economic, and political power. Implementing effective self-care interventions may be effective for women who have experienced HDP, as they are at high risk of developing CVD. Current guidelines recommend adopting a healthy lifestyle and undergoing regular follow-ups for cardiovascular risk assessment and management to reduce the risk of developing CVD ^52,53^. However, the exact mechanism by which self-care practices reduce the risk of CVD is not fully understood. One proposed mechanism suggests that self-care practices have a cardioprotective effect, such as minimising inflammation or avoiding risky pharmacological treatment ^54^.

- 1. Rationale

Early prevention strategies might be necessary to mitigate the risk of developing CVD in individuals with a history of HDP. Currently, there are no WHO self-care guidelines on how to minimise CVD risks in people with a history of HDP ^55^. Although existing and emerging reviews on lifestyle interventions focus on reducing CVD risks in women with previous HDP, no reviews have specifically evaluated self-care interventions ^56–61^. Self-care interventions encompass a wide range of approaches, including health literacy, regular physical activity, understanding risks and practices to mitigate them, being aware of dangers and taking action ^62^. The effectiveness of self-care interventions may vary depending on the target population or the setting in which they occur. For postpartum women, maintaining self-care practices can be challenging due to the demands of motherhood, financial limitations, and the feelings of guilt for accepting help and prioritising their well-being ^63^. Additionally, adherence to social and cultural practices such as activity and dietary restrictions influence women’s care practices after childbirth and in the long term ^64^. Due to these challenges and the persistent risk of HDP, it is crucial to identify the most effective self-care interventions for women with a history of HDP, regardless of their postpartum period. Therefore, we aim to synthesise the available evidence on the effectiveness of self-care interventions for the prevention of cardiovascular diseases in women with previous HDP. The findings have public health implications for reducing the burden of cardiovascular diseases. It can inform clinical practice and policies on the long-term management of individuals with previous HDP.

1. **Objectives**

We aim to synthesise the available evidence on the effectiveness of self-care interventions for the prevention of cardiovascular diseases in women with previous HDP.

*Review question*

Compared to no intervention, usual or standard care, do self-care interventions reduce the incidence of cardiovascular events, and chronic hypertension and reduce risk factors of CVD in women with a history of HDP?

1. **Methods**

We will conduct a systematic review of randomised and non-randomised trials that assess the effectiveness of self-care interventions to reduce the risk of cardiovascular disease in women with previous HDP. When reporting our findings, we will adhere to the PRISMA guidelines ^65^ and the Cochrane Handbook for Systematic Reviews ^1^. To achieve this, we will develop a search strategy to identify relevant studies from various publication databases, screen the literature to find studies that meet the eligibility criteria, locate, and evaluate the full texts of potential studies, extract data from the eligible studies, assess the risk of bias in each study, and synthesise the results.

- 1. Eligibility criteria
     1. Population

Non-pregnant women with a history of HDP, regardless of the duration of the last pregnancy.

- - 1. Intervention

For the purpose of this review, a self-care intervention is defined as any tool, strategy or resource designed to promote or facilitate self-care among women who have had
HDP, regardless of the duration from the last pregnancy, single or combined with other self-care interventions, to improve the coverage and quality of healthcare services and/or enhance their well-being, health, and care experiences.

- - 1. Comparison

The comparison is no intervention, usual or standard care.

- - 1. Outcomes

Outcomes are selected based on the recommended core components of CVD ^66,67^ and common self-care intervention outcomes for people with HDP.

- - - 1. *Primary outcomes*
  - Cardiovascular event (such as stroke, or myocardial infarction, or heart failure), as defined by the authors.
  - Chronic hypertension, as defined by the authors.
    - 1. *Secondary outcomes*
  - Modifiable
- BMI
- Diabetes/Impaired glucose tolerance
- Blood pressure (mean diastolic and systolic blood pressure)
- Lipid levels
- Physical activity, as measured by the study
- Dietary intake as measured by the study
- Breastfeeding status (e.g., self-reported)
- Psychological status (Health-related quality of life, depression and anxiety assessed using a validated score)
- Smoking status (e.g., self-reported, salivary cotinine)
- Caffeine use status
- Alcohol use status (e.g., self-reported, referral to alcohol rehab)
- Illicit drug use (e.g., self-reported, referral to drug rehab)
- Social harms (e.g., stigma, intimate partner violence
- Autonomy (e.g., self-efficacy, empowerment)
- Antihypertensive medication requirement
- Other as defined by the study
  - Compliance of patients or participant retention with long-term follow-up, as measured by attendance records.
    1. Study design

All randomised and non-randomised trials (including cross-over trials, cluster randomised and quasi-experimental trials) administering self-care interventions to reduce the risk of cardiovascular disease will be eligible, provided there is an appropriate comparator group. The following studies will be excluded:

1. Trials that combine self-care interventions with other interventions (e.g., therapeutic interventions) without a comparison group that only receives the other intervention.
2. Trials that compare various self-care interventions without the use of a control group.
3. Trials in which caregivers (family members or partners) carried out self-care for the women with HDP.
4. Trials with women with pre-existing CVD (such as pre-existing hypertension, renal failure, and cardiac disease) before the index pregnancy.
5. Scoping reviews, meta-analyses, systematic reviews, overviews of systematic reviews, observational cohort studies, cross-sectional studies, case series, case studies, case reports, dissertations and theses, reports published as abstracts only, and qualitative studies.
6. Interventions primarily based on another population, such as gestational diabetes or high BMI, which incidentally reported on women with previous HDP.
   1. Electronic databases and searching methods

We will systematically search five electronic databases, including MEDLINE, Embase, CINAHL, PsycINFO, and the Cochrane Library, for studies published up until the current date. There will be no restrictions on the language or date of publication. We will consult an information specialist to optimise the search strategy and manually search the included articles' references for potentially eligible studies.

- 1. Study selection and screening

Two reviewers will independently assess articles identified through the database search for eligibility at both the title/abstract and full-text screening stages. Any disagreements will be resolved by discussion and consultation with a third reviewer. Articles published in languages other than English will be screened using a translation app tool, or a native speaker will be consulted.

- 1. Trial integrity assessment

All trials meeting the inclusion criteria after full-text screening will undergo research integrity assessment using a published research integrity assessment tool ^68^. This approach will ensure that any problematic studies are identified and managed transparently to avoid misleading findings.

- 1. Data extraction

Two reviewers will independently extract data using a standardised extraction tool in Covidence. Any discrepancies will be resolved by discussion or with a third reviewer. If necessary, we will contact the authors of the included trials for further information.

The data extraction form will include the following:

- Study characteristics
- Authors, title, year of publication, and location of study
- Sample size, study design, setting (secondary or tertiary healthcare facility), year(s) of study enrolment.
- Definitions used for self-care, risk of CVD, hypertensive disorders during pregnancy, etc.
- Participant characteristics
- Eligibility criteria for the study population
- Postpartum year(s) at enrolment/ year(s) after the last delivery at enrolment
- Number of children
- Breastfeeding (breastfeeding status or history of breastfeeding)
- Comorbidities in pregnancy such as gestational diabetes, thyroid disorders, increased BMI.
- Pre-existing medical conditions such as type 1 or 2 diabetes, hypercholesterolemia, asthma, HIV, etc.
- Intervention characteristics
- Type of intervention
- Duration of intervention
- Comparison or control group
- Primary and secondary outcomes as outlined above.
  1. Quality assessment

The quality of identified randomised controlled trials will be assessed using the revised Cochrane risk-of-bias tool for randomised trials (RoB 2) ^69^, and the ROBINS-I tool will be used for non-randomised trials ^70^.

- 1. Reporting measures

Findings will be reported per study. We plan to conduct a meta-analysis for each outcome where sufficient data are available.

- For incidence prevalence outcomes, data shall be reported as number (%)
- Continuous data will be reported as mean (SD) or median (IQR)
  - 1. Missing data

For included trials, we will report levels of attrition. We will use sensitivity analysis to explore the impact of including trials with high levels of missing data on the overall assessment. Funnel plots will be used to investigate publication bias.

- - 1. Heterogeneity assessment

We will assess statistical heterogeneity in meta-analyses using the T² (tau-squared), I² and Chi² statistics. We will regard heterogeneity as substantial if T² was greater than zero and either I² was greater than 30% or there was a low P-value (less than 0.10) in the Chi² test for heterogeneity.

- 1. Data synthesis

We will carry out statistical analysis using STATA meta-analysis software. We will use random-effect meta-analysis for combining data where it was reasonable to assume that studies were estimating prevalence in sufficiently similar populations. We will conduct meta-analyses from randomised and non-randomised interventional trials for all outcomes with sufficient data.

For both outcomes, if clinical, methodological, or other heterogeneity is sufficient to expect that the underlying incidence will differ between studies, or if substantial statistical heterogeneity is detected, we will use a random-effects analysis to produce an overall summary). If an average prevalence across trials is not clinically meaningful, we will not combine heterogeneous studies. If we use random-effects analyses, the results will be presented as the risk ratio (for binomial variables), mean difference (for continuous variables) and their 95% confidence intervals, and the estimates of T² and I². If data cannot be analysed using meta-analysis, we will present a descriptive analysis of the findings.

- - 1. Subgroup analysis

We planned to conduct the following *a priori* subgroup analyses on all outcomes (provided sufficient data exists).

- Setting: low- and middle-income country vs high-income country
- Preeclampsia vs other HDPs
- Type of intervention (lifestyle changes, self-monitoring or education, etc.)
- Single or combined interventions
- Postpartum period at enrolment
  1. Rating the certainty of evidence

Two authors will independently rate the certainty of evidence included in the review by using Grading of Recommendations, Assessment, Development, and Evaluations (GRADE) ^71^. Any disagreements will be resolved through discussion or consultation with a third author.

**Reference for protocol:**

1. Higgins J, Thomas J, Chandler J, Cumpston M, Li T, Page M, et al. Cochrane Handbook for Systematic Reviews of Interventions version 6.4 (updated August 2023) [Internet]. 2023 [cited 2023 Aug 22]. Available from: https://training.cochrane.org/handbook/current

2. Follmann D, Elliott P, Suh I, Cutler J. Variance imputation for overviews of clinical trials with continuous response. J Clin Epidemiol. 1992 Jul;45(7):769–73.

3. Berlim MT, McGirr A, Rodrigues dos Santos N, Tremblay S, Martins R. Efficacy of theta burst stimulation (TBS) for major depression: An exploratory meta-analysis of randomized and sham-controlled trials. Journal of Psychiatric Research. 2017 Jul 1;90:102–9.

4. Papadopoulos VP, Apergis N, Filippou DK. Nocturia in CPAP-Treated Obstructive Sleep Apnea Patients: a Systematic Review and Meta-Analysis. SN Compr Clin Med. 2020 Dec 1;2(12):2799–807.

5. Yagiz G, Akaras E, Kubis HP, Owen JA. The Effects of Resistance Training on Architecture and Volume of the Upper Extremity Muscles: A Systematic Review of Randomised Controlled Trials and Meta-Analyses. Applied Sciences. 2022 Jan;12(3):1593.

6. Mavridis D, White IR. Dealing with missing outcome data in meta-analysis. Research Synthesis Methods. 2020;11(1):2–13.

7. White IR, Higgins J. Meta-Analysis with missing data. Meta-Analysis in Stata: An Updated Collection from the Stata Journal, Second Edition [Internet]. STATA. 2016 [cited 2024 Jun 7]. Available from: https://www.stata.com/bookstore/meta-analysis-in-stata/

8. Mavridis D, Chaimani A. Allowing for uncertainty due to missing outcome data in meta-analysis [Internet]. Cochrane. 2018 [cited 2024 Jun 7]. Available from: https://training.cochrane.org/resource/allowing-uncertainty-due-missing-outcome-data-meta-analysis

9. Kahale LA, Khamis AM, Diab B, Chang Y, Lopes LC, Agarwal A, et al. Potential impact of missing outcome data on treatment effects in systematic reviews: imputation study. BMJ. 2020 Aug 26;370:m2898.

10. Meng Z, Wang J, Lin L, Wu C. Sensitivity analysis with iterative outlier detection for systematic reviews and meta-analyses. Statistics in Medicine. 2024;43(8):1549–63.

11. Cumpston M, Li T, Page MJ, Chandler J, Welch VA, Higgins JP, et al. Updated guidance for trusted systematic reviews: a new edition of the Cochrane Handbook for Systematic Reviews of Interventions. Cochrane Database Syst Rev. 2019 Oct 3;2019(10):ED000142.

12. Arkerson BJ, Finneran MM, Harris SR, Schnorr J, McElwee ER, Demosthenes L, et al. Remote Monitoring Compared With In-Office Surveillance of Blood Pressure in Patients With Pregnancy-Related Hypertension. Obstet Gynecol. 2023 Oct;142(4):855–61.

13. Cairns AE, Tucker KL, Leeson P, Mackillop LH, Santos M, Velardo C, et al. Self-Management of Postnatal Hypertension: The SNAP-HT Trial. Hypertension (0194911X). 2018 Aug;72(2):425–32.

14. Ekawati E, Setyowati S, Budiati T. “Sehati” health education to improve physical and psychological adaptation of the postpartum women having pre-eclampsia. Enfermeria Clinica. 2019 Jan 1;29:199–204.

15. Hauspurg A, Seely EW, Rich-Edwards J, Hayduchok C, Bryan S, Roche AT, et al. Postpartum home blood pressure monitoring and lifestyle intervention in overweight and obese individuals the first year after gestational hypertension or pre-eclampsia: A pilot feasibility trial. BJOG. 2023 Jun;130(7):715–26.

16. Hirshberg A, Downes K, Srinivas S. Comparing standard office-based follow-up with text-based remote monitoring in the management of postpartum hypertension: a randomised clinical trial. BMJ Quality & Safety. 2018 Nov;27(11):871–7.

17. Hoppe KK, Thomas N, Zernick M, Zella JB, Havighurst T, Kim K, et al. Telehealth with remote blood pressure monitoring compared with standard care for postpartum hypertension. Am J Obstet Gynecol. 2020 Oct;223(4):585–8.

18. Hutchesson MJ, Taylor R, Shrewsbury VA, Vincze L, Campbell LE, Callister R, et al. Be Healthe for Your Heart: A Pilot Randomized Controlled Trial Evaluating a Web-Based Behavioral Intervention to Improve the Cardiovascular Health of Women with a History of Preeclampsia. Int J Environ Res Public Health. 2020 Aug 10;17(16):5779.

19. Kitt JA, Fox RL, Cairns AE, Mollison J, Burchert HH, Kenworthy Y, et al. Short-Term Postpartum Blood Pressure Self-Management and Long-Term Blood Pressure Control: A Randomized Controlled Trial. Hypertension (0194911X). 2021 Aug;78(2):469–79.

20. Kitt J, Fox R, Frost A, Shanyinde M, Tucker K, Bateman PA, et al. Long-Term Blood Pressure Control After Hypertensive Pregnancy Following Physician-Optimized Self-Management: The POP-HT Randomized Clinical Trial. JAMA. 2023 Nov 28;330(20):1991–9.

21. Lewey J, Murphy S, Zhang D, Putt ME, Elovitz MA, Riis V, et al. Effectiveness of a Text-Based Gamification Intervention to Improve Physical Activity Among Postpartum Individuals With Hypertensive Disorders of Pregnancy: A Randomized Clinical Trial. JAMA Cardiol. 2022 Jun 1;7(6):591–9.

22. Muijsers HEC, Wu P, van der Heijden OWH, Wijnberger LDE, van Bijsterveldt C, Buijs C, et al. Home blood pressure monitoring detects unrevealed hypertension in women with a history of preeclampsia: Results of the BP-PRESELF study. Am J Prev Cardiol. 2022 Nov 11;12:100429.

23. Nicklas JM, Pyle L, Soares A, Leiferman JA, Bull SS, Tong S, et al. The Fit After Baby randomized controlled trial: An mHealth postpartum lifestyle intervention for women with elevated cardiometabolic risk. PLOS ONE. 2024 Jan 9;19(1):e0296244.

24. Parfenova M, Côté AM, Cumyn A, Pesant MH, Champagne M, Roy-Lacroix MÈ, et al. Impact of an Educational Pamphlet on Knowledge About Health Risks After Hypertensive Disorders of Pregnancy: A Randomized Trial. J Obstet Gynaecol Can. 2021 Feb;43(2):182–90.

25. Rich-Edwards JW, Stuart JJ, Skurnik G, Roche AT, Tsigas E, Fitzmaurice GM, et al. Randomized Trial to Reduce Cardiovascular Risk in Women with Recent Preeclampsia. Journal of Women’s Health (15409996). 2019 Nov;28(11):1493–504.

26. Riemer M, Schulze S, Wagner L, Richter M, Ayerle G, Simm A, et al. Cardiovascular Risk Reduction in Women Following Hypertensive Disorders of Pregnancy – a Prospective, Randomised, Controlled Interventional Study. Geburtshilfe Frauenheilkd. 2021 Aug;81(8):966–78.

27. Wang TL, Quinn BA, Hart R, Wiener AA, Facco FL, Simhan HN, et al. The effect of a neonatal sleep intervention on maternal postpartum hypertension: a randomized trial. Am J Obstet Gynecol MFM. 2024 Feb;6(2):101239.

28. Magee LA, Brown MA, Hall DR, Gupte S, Hennessy A, Karumanchi SA, et al. The 2021 International Society for the Study of Hypertension in Pregnancy classification, diagnosis & management recommendations for international practice. Pregnancy Hypertens. 2022 Mar;27:148–69.

30. Report of the National High Blood Pressure Education Program Working Group on High Blood Pressure in Pregnancy. Am J Obstet Gynecol. 2000 Jul;183(1):S1–22.

31. Bulletins—Obstetrics AC of O and GC on P. ACOG Practice Bulletin No. 203: Chronic Hypertension in Pregnancy. Obstet Gynecol. 2019 Jan;133(1):e26–50.

32. Hutcheon JA, Lisonkova S, Joseph KS. Epidemiology of pre-eclampsia and the other hypertensive disorders of pregnancy. Best Practice & Research Clinical Obstetrics & Gynaecology. 2011 Aug 1;25(4):391–403.

33. Wang W, Xie X, Yuan T, Wang Y, Zhao F, Zhou Z, et al. Epidemiological trends of maternal hypertensive disorders of pregnancy at the global, regional, and national levels: a population‐based study. BMC Pregnancy and Childbirth. 2021 May 8;21(1):364.

34. Jiang L, Tang K, Magee LA, von Dadelszen P, Ekeroma A, Li X, et al. A global view of hypertensive disorders and diabetes mellitus during pregnancy. Nat Rev Endocrinol. 2022;18(12):760–75.

35. Ronsmans C, Graham WJ. Maternal mortality: who, when, where, and why. The Lancet. 2006 Sep 30;368(9542):1189–200.

36. Li F, Wang T, Chen L, Zhang S, Chen L, Qin J. Adverse pregnancy outcomes among mothers with hypertensive disorders in pregnancy: A meta-analysis of cohort studies. Pregnancy Hypertension. 2021 Jun 1;24:107–17.

37. Umesawa M, Kobashi G. Epidemiology of hypertensive disorders in pregnancy: prevalence, risk factors, predictors and prognosis. Hypertens Res. 2017 Mar;40(3):213–20.

38. Duffy J, Cairns AE, Richards-Doran D, van ’t Hooft J, Gale C, Brown M, et al. A core outcome set for pre-eclampsia research: an international consensus development study. BJOG. 2020 Nov;127(12):1516–26.

39. Ying W, Catov JM, Ouyang P. Hypertensive Disorders of Pregnancy and Future Maternal Cardiovascular Risk. J Am Heart Assoc. 2018 Aug 28;7(17):e009382.

40. Sukmanee J, Liabsuetrakul T. Risk of future cardiovascular diseases in different years postpartum after hypertensive disorders of pregnancy: A systematic review and meta-analysis. Medicine (Baltimore). 2022 Jul 29;101(30):e29646.

41. World Health Organization. Cardiovascular diseases (CVDs) [Internet]. 2021 [cited 2023 Aug 30]. Available from: https://www.who.int/news-room/fact-sheets/detail/cardiovascular-diseases-(cvds)

42. Romundstad PR, Magnussen EB, Smith GD, Vatten LJ. Hypertension in pregnancy and later cardiovascular risk: common antecedents? Circulation. 2010 Aug 10;122(6):579–84.

43. Stuart JJ, Tanz LJ, Missmer SA, Rimm EB, Spiegelman D, James-Todd TM, et al. Hypertensive Disorders of Pregnancy and Maternal Cardiovascular Disease Risk Factor Development: An Observational Cohort Study. Ann Intern Med. 2018 Aug 21;169(4):224–32.

44. Odukoya SA, Moodley J, Naicker T. Current Updates on Pre-eclampsia: Maternal and Foetal Cardiovascular Diseases Predilection, Science or Myth? : Future cardiovascular disease risks in mother and child following pre-eclampsia. Curr Hypertens Rep. 2021 Mar 10;23(3):16.

45. Di Giosia P, Passacquale G, Petrarca M, Giorgini P, Marra AM, Ferro A. Gender differences in cardiovascular prophylaxis: Focus on antiplatelet treatment. Pharmacological Research. 2017 May 1;119:36–47.

46. Vogel B, Acevedo M, Appelman Y, Bairey Merz CN, Chieffo A, Figtree GA, et al. The Lancet women and cardiovascular disease Commission: reducing the global burden by 2030. The Lancet. 2021 Jun;397(10292):2385–438.

47. Magee LA, Smith GN, Bloch C, Côté AM, Jain V, Nerenberg K, et al. Guideline No. 426: Hypertensive Disorders of Pregnancy: Diagnosis, Prediction, Prevention, and Management. Journal of Obstetrics and Gynaecology Canada. 2022 May 1;44(5):547-571.e1.

48. World Health Organization. Self-care interventions for health [Internet]. World Health Organization. 2022 [cited 2023 Aug 17]. Available from: https://www.who.int/news-room/fact-sheets/detail/self-care-health-interventions

49. Riegel B, Jaarsma T, Strömberg A. A Middle-Range Theory of Self-Care of Chronic Illness. Advances in Nursing Science. 2012 Sep;35(3):194.

50. van den Heuvel JFM, Lely AT, Huisman JJ, Trappenburg JCA, Franx A, Bekker MN. SAFE@HOME: Digital health platform facilitating a new care path for women at increased risk of preeclampsia - A case-control study. Pregnancy Hypertens. 2020 Oct;22:30–6.

51. Riegel B, Westland H, Freedland KE, Lee CS, Stromberg A, Vellone E, et al. Operational definition of self-care interventions for adults with chronic illness. International Journal of Nursing Studies. 2022 May 1;129:104231.

52. Lowe SA, Bowyer L, Lust K, McMahon LP, Morton M, North RA, et al. SOMANZ guidelines for the management of hypertensive disorders of pregnancy 2014. Australian and New Zealand Journal of Obstetrics and Gynaecology. 2015;55(5):e1–29.

53. Brown MA, Magee LA, Kenny LC, Karumanchi SA, McCarthy FP, Saito S, et al. Hypertensive Disorders of Pregnancy: ISSHP Classification, Diagnosis, and Management Recommendations for International Practice. Hypertension. 2018 Jul;72(1):24–43.

54. Lee CS, Tkacs NC, Riegel B. The Influence of Heart Failure Self-Care on Health Outcomes: Hypothetical Cardioprotective Mechanisms. J Cardiovasc Nurs. 2009;24(3):179–89.

55. World Health Organization. WHO guideline on self-care interventions for health and well-being, 2022 revision [Internet]. 2022 [cited 2023 Aug 17]. Available from: https://app.magicapp.org/#/guideline/5512

56. Lui NA, Jeyaram G, Henry A. Postpartum Interventions to Reduce Long-Term Cardiovascular Disease Risk in Women After Hypertensive Disorders of Pregnancy: A Systematic Review. Frontiers in Cardiovascular Medicine [Internet]. 2019 [cited 2023 Aug 17];6. Available from: https://www.frontiersin.org/articles/10.3389/fcvm.2019.00160

57. Behnam S, Timmesfeld N, Arabin B. Lifestyle Interventions to Improve Pregnancy Outcomes: a Systematic Review and Specified Meta-Analyses. Geburtshilfe Frauenheilkd. 2022 Nov 3;82(11):1249–64.

58. Simpson G, Atkinson J, Lindquist A. Post-partum lifestyle interventions and follow-up for cardiovascular health in women with hypertensive disorders of pregnancy: a systematic review of Australian clinical guidelines [Internet]. PROSPERO International prospective register of systematic reviews. 2022 [cited 2023 Aug 17]. Available from: https://www.crd.york.ac.uk/PROSPERO/display_record.php?RecordID=328892

59. Taylor F, Lang S, Moran L. Lifestyle interventions in postpartum women with adverse pregnancy outcomes: a systematic review [Internet]. PROSPERO International prospective register of systematic reviews. 2022 [cited 2023 Aug 17]. Available from: https://www.crd.york.ac.uk/PROSPERO/display_record.php?RecordID=349071

60. Halligan J, Whelan M, Farmer A. Reducing cardiovascular risk and preventing diabetes following gestational diabetes: a systematic review of interventions including a digital or telemedicine component [Internet]. PROSPERO International prospective register of systematic reviews. 2019 [cited 2023 Aug 17]. Available from: https://www.crd.york.ac.uk/PROSPERO/display_record.php?RecordID=145051

61. Yeh PT, Rhee DK, Kennedy CE, Zera CA, Lucido B, Tunçalp Ö, et al. Self-monitoring of blood pressure among women with hypertensive disorders of pregnancy: a systematic review. BMC Pregnancy and Childbirth. 2022 May 31;22(1):454.

62. International Self-Care Foundation. The Seven Pillars of Self-Care [Internet]. ISF. [cited 2023 Aug 17]. Available from: https://isfglobal.org/practise-self-care/the-seven-pillars-of-self-care/

63. Barkin JL, Wisner KL. The role of maternal self-care in new motherhood. Midwifery. 2013 Sep;29(9):1050–5.

64. Udoji A. Culturally competent care in postpartum period. In 2014 [cited 2023 Sep 28]. Available from: https://www.semanticscholar.org/paper/Culturally-competent-care-in-postpartum-period.-Udoji/78c73c5063cc3644c8b31b320e9800256621aab6

65. Page MJ, McKenzie JE, Bossuyt PM, Boutron I, Hoffmann TC, Mulrow CD, et al. The PRISMA 2020 statement: an updated guideline for reporting systematic reviews. Systematic Reviews. 2021 Mar 29;10(1):89.

66. Cowie A, Buckley J, Doherty P, Furze G, Hayward J, Hinton S, et al. Standards and Core Components for Cardiovascular Disease Prevention and Rehabilitation. Heart. 2019 Jan 30;105:1–6.

67. Woodruffe S, Neubeck L, Clark RA, Gray K, Ferry C, Finan J, et al. Australian Cardiovascular Health and Rehabilitation Association (ACRA) Core Components of Cardiovascular Disease Secondary Prevention and Cardiac Rehabilitation 2014. Heart, Lung and Circulation. 2015 May 1;24(5):430–41.

68. Weibel S, Popp M, Reis S, Skoetz N, Garner P, Sydenham E. Identifying and managing problematic trials: a Research Integrity Assessment (RIA) tool for randomized controlled trials in evidence synthesis [Internet]. medRxiv; 2022 [cited 2023 Aug 29]. p. 2022.05.31.22275756. Available from: https://www.medrxiv.org/content/10.1101/2022.05.31.22275756v1

69. Sterne JAC, Savović J, Page MJ, Elbers RG, Blencowe NS, Boutron I, et al. RoB 2: a revised tool for assessing risk of bias in randomised trials. BMJ. 2019 Aug 28;366:l4898.

70. Sterne JA, Hernán MA, Reeves BC, Savović J, Berkman ND, Viswanathan M, et al. ROBINS-I: a tool for assessing risk of bias in non-randomised studies of interventions. BMJ. 2016 Oct 12;355:i4919.

71. Schünemann H, Brożek J, Guyatt G, Oxman A. GRADE handbook [Internet]. Cochrane. 2013 [cited 2023 Aug 29]. Available from: https://gdt.gradepro.org/app/handbook/handbook.html
